# Behaviorally Activated mRNA Expression Profiles Produce Signatures of Learning and Enhanced Inhibition in Aged Rats with Preserved Memory

**DOI:** 10.1371/journal.pone.0083674

**Published:** 2013-12-13

**Authors:** Rebecca P. Haberman, Carlo Colantuoni, Ming Teng Koh, Michela Gallagher

**Affiliations:** 1 Department of Psychological and Brain Sciences, The Johns Hopkins University, Baltimore, Maryland, United States of America; 2 Lieber Institute for Brain Development, and Department of Neurology, Johns Hopkins School of Medicine, Baltimore, Maryland, United States of America; Nathan Kline Institute and New York University School of Medicine, United States of America

## Abstract

Aging is often associated with cognitive decline, but many elderly individuals maintain a high level of function throughout life. Here we studied outbred rats, which also exhibit individual differences across a spectrum of outcomes that includes both preserved and impaired spatial memory. Previous work in this model identified the CA3 subfield of the hippocampus as a region critically affected by age and integral to differing cognitive outcomes. Earlier microarray profiling revealed distinct gene expression profiles in the CA3 region, under basal conditions, for aged rats with intact memory and those with impairment. Because prominent age-related deficits within the CA3 occur during neural encoding of new information, here we used microarray analysis to gain a broad perspective of the aged CA3 transcriptome under activated conditions. Behaviorally-induced CA3 expression profiles differentiated aged rats with intact memory from those with impaired memory. In the activated profile, we observed substantial numbers of genes (greater than 1000) exhibiting increased expression in aged unimpaired rats relative to aged impaired, including many involved in synaptic plasticity and memory mechanisms. This unimpaired aged profile also overlapped significantly with a learning induced gene profile previously acquired in young adults. Alongside the increased transcripts common to both young learning and aged rats with preserved memory, many transcripts behaviorally-activated in the current study had previously been identified as repressed in the aged unimpaired phenotype in basal expression. A further distinct feature of the activated profile of aged rats with intact memory is the increased expression of an ensemble of genes involved in inhibitory synapse function, which could control the phenotype of neural hyperexcitability found in the CA3 region of aged impaired rats. These data support the conclusion that aged subjects with preserved memory recruit adaptive mechanisms to retain tight control over excitability under both basal and activated conditions.

## Introduction

Significant variability characterizes cognitive outcomes in aging populations. Many individuals experience cognitive difficulties that typically emerge as mild memory deficits but can progress to greater impairment than expected for a person’s age and eventually to clinical dementia. However, a significant proportion of the elderly maintain a high level of cognitive function, including intact memory, well into old age [1,2]. Two concepts predominate in current views on the basis for preserved cognitive function in aging: Brain maintenance and cognitive reserve. Brain maintenance refers to the idea that intact capacity depends on abrogating or delaying age-related changes in brain systems that typically lead to decline [2]. By that view, a high level of cognitive function has a similar basis in brain function, irrespective of chronological age. The idea of cognitive reserve centers around a robust capacity of brain networks and cognitive resources to compensate for the neurobiological effects of aging on the brain. Reserve varies among individuals, may be susceptible to environmental influences, and results in varied aging outcomes [3]. While evidence for both of these perspectives can be found in the literature on human aging [2], direct examination of brain neurophysiology, which can be best performed in animal models, can provide significant additional insight into the basis for individual differences in aging. 

Aged Long Evans rats exhibit naturally occurring individual differences in cognition, including rats with impairment and aged cohorts with proficient performance on behavioral tasks assessing spatial memory [4]. Optimal performance in these assessments used to characterize individual differences depends upon intact function of the medial temporal lobe (MTL), a system that also supports episodic memory in humans, representing one of the earliest affected domains in age-related cognitive decline [5]. The Long Evans rodent model has provided a particularly extensive neurobiological characterization of the memory-impaired phenotype [6].

Accumulated work in this model has focused attention on the CA3 subfield of the hippocampus, which exhibits age- and memory-related neurophysiological alterations affecting circuit properties. Reduced perforant path synaptic innervation of the CA3 region, which provides the major cortical input to this region, and increased firing rates of CA3 pyramidal neurons are associated with specific neural encoding deficits and impaired behavioral performance in aged rats [7,8]. Correlates of these alterations have been identified in human studies using analogous cognitive assessments and functional magnetic resonance imaging (fMRI). Aged humans and individuals diagnosed with amnestic mild cognitive impairment (aMCI), a condition in which memory is worse than expected for a person’s age, show increased CA3/DG BOLD signal when performing a memory task that places demands on the encoding properties of these circuits [9,10]. Targeting CA3 hyperactivity with a pharmacological therapy, first in aged impaired rats and then in both a mouse model of Alzheimer’s disease and in humans with aMCI, has demonstrated improved memory performance in all three settings [11-13] emphasizing the translational value of this model. 

Recently we examined gene expression profiles in the CA3 hippocampal subfield of behaviorally characterized young and aged rats under basal (homecage) conditions [14]. In aged impaired rats, we detected mRNA signatures that could contribute to neurophysiological features noted above. In particular, we observed reduced expression in aged rats with behavioral impairment of genes underlying synaptic inhibition, consistent with increased neuronal firing rates recorded from principal CA3 pyramidal cells. Furthermore, using principal component analysis to provide a broad perspective on individual differences among the aged rats, we found that CA3 gene expression profiles not only distinguished aged rats with intact memory from aged impaired subjects but also distinguished the unimpaired aged rats from young. Indeed, we identified and then independently confirmed specific genes with distinctive expression in aged rats with intact cognition (i.e. genes that were differentially expressed in aged unimpaired relative to both young and aged impaired), suggesting that preserved cognition is not simply attributable to brain maintenance but instead might involve mechanisms to actively oppose impairment-related alterations in key memory circuits.

Although gene expression profiles of the CA3 region in the basal condition were notable for distinguishing the aged rat phenotypes with respect to cognitive outcome, prominent age-related deficits in neural function within the CA3 appear during active encoding of new information [6,8]. New learning induces dynamic regulation of hippocampal gene expression, which is critical to long-term synaptic plasticity and long-term memory [15-17]. We previously demonstrated that this requirement applies to the CA3 subfield in young rats trained in a spatial memory task [18]. Here we extend that approach to employ gene expression profiling of the CA3 region in aged rats characterized for intact or impaired memory using the same behavioral induction protocol previously employed with young adults [18]. The distinctive expression patterns exhibited by aged intact rats, compared with our previously published data on young adults under the same task conditions, allows a further examination of the brain maintenance hypothesis. 

## Results

### Behavioral characterization and induction training of aged rats

All rats (aged 24 months) were assessed in a standardized protocol used for behavioral characterization in this model to generate a learning index (LI) for each rat (see methods and [4]). The protocol consisted of 8 days of hidden-platform watermaze training with interpolated probe trials. The LI is a weighted score derived from probe trial performance and is a robust measure of individual differences in spatial memory using this model [4,19,20]. Aged rats performing within the normative range of young adults at 6 months of age (LI <240) were designated aged unimpaired (AU) while those performing outside the normal range of young adults (LI >240) were considered aged impaired (AI). Sixteen AU and 16 AI rats were used in the current study. 

**Figure 1:**
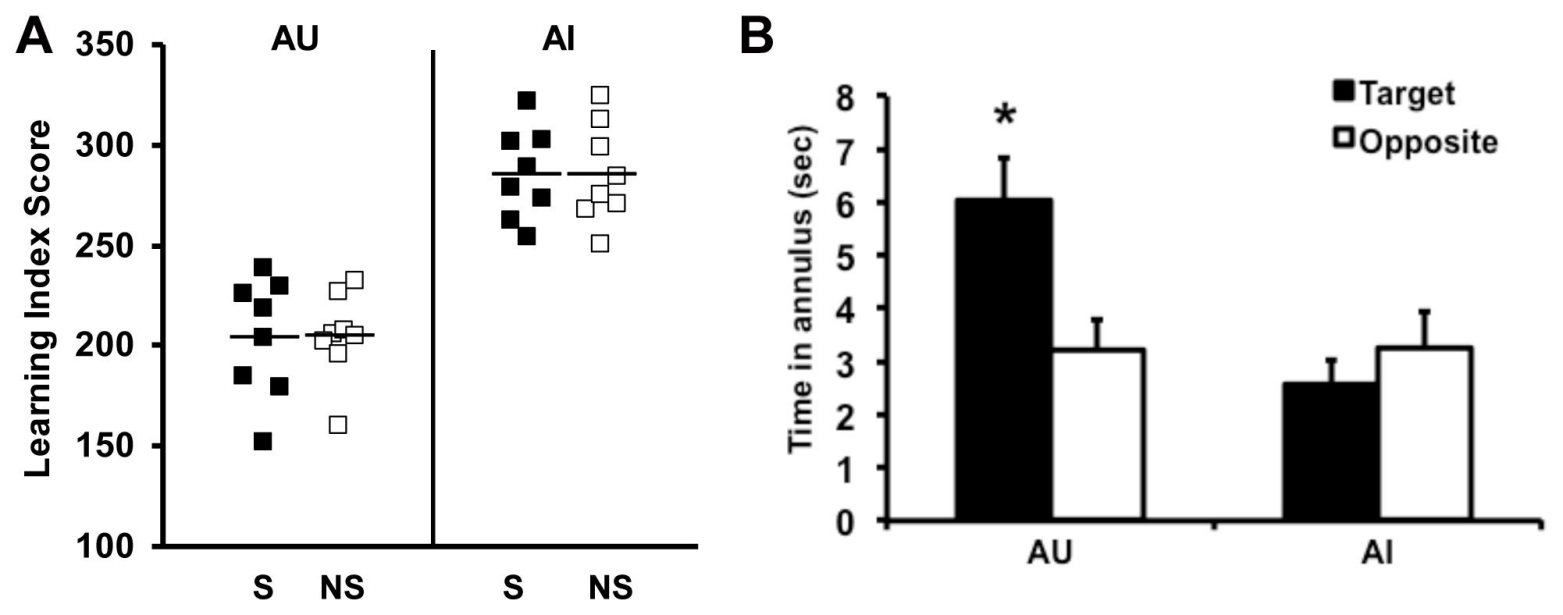
Behavioral characterization and induction training. **A**. Behaviorally characterized aged unimpaired (AU) and aged impaired (AI) rats were assigned to spatial (S) or non-spatial (NS) training conditions. S and NS groups were matched for learning index within each cognitive phenotype. Group mean is represented by horizontal dash. N=8 per group. **B**. During a probe trial administered 1 hour after the end of induction training, AU rats trained in the spatial condition showed a search bias for the platform location. For AU-S rats, time spent in target annulus was significantly greater than time spent in the opposite annulus (*, paired t-test, p=0.039) while no significant spatial bias was seen for AI-S rats (p=0.258). Error bars represent SEM.

Approximately 1 month after the completion of initial behavioral characterization, all AI and AU rats were trained in a protocol that we previously used to induce gene expression in young adult rats [18]. This training occurred in a novel environment with the water maze located in a different building. In a single session, all rats were given 8 trials (8 min. inter-trial interval) to swim to a visible platform. AU and AI rats were assigned to one of two versions of the 8-trial protocol, designated spatial and non-spatial, such that half the rats performed the task in the presence of prominent extra-maze cues surrounding the pool with a fixed platform location (spatial, S), which rendered explicit spatial information relevant to task performance but not required to escape proficiently by swimming to the visible platform. The other half performed a control task (designated non spatial, NS) in which the platform was moved to a different location on each trial to make its position in the environment irrelevant in the task. In the control task the prominent extramaze cues were also removed to further minimize spatial information in the novel environment. In assigning rats across S or NS conditions (shown in Figure 1A), learning index scores were matched as confirmed by statistical analysis (a 2 x 2 ANOVA indicated a main effect for cognitive phenotype [AU LIs were lower than those for AI, F(1, 28), = 84.17, p = 0.001] but no interaction between phenotype and training condition). Because the platform was visible across both training conditions in this induction protocol, rats spent an equivalent amount of time in the pool as measured by escape latency [F(3, 31) = 0.817. p = 0.495], thus equating motoric output and stress conditions across groups, irrespective of cognitive status or training condition. Statistical analysis of the training trial data also showed that aged rats improved their performance in swimming to the visible platform across training trials [F(1,28) = 15.55, p=0.001], and there were no differences as a function of aged phenotype (AU vs AI), training protocol (S vs NS) or interactions among those factors (data not shown). 

One hour after training (and just prior to sacrifice) rats were given a probe test in the absence of the platform to assess memory for the escape location. AU rats in the S training condition spent significantly more time in the immediate vicinity of the target location compared to a control area in the opposite quadrant of the pool, demonstrating a spatial bias, [t(7) = 2.52, p=0.039 (Figure 1B)]. In contrast, no spatial bias was seen for the AI rats [t(7) = 1.23, p=0.258] consistent with the initial behavioral characterization for those rats. Thus AU rats in the current experiment, similar to young adult rats trained in the same protocol [18], exhibited spatial learning that was incidental to task contingencies when a visible platform remained in the same escape location. As expected, rats trained in the NS condition, irrespective of original behavioral characterization, showed no spatial bias to the designated target platform location used in the spatial version of the induction protocol (data not shown). 

### Microarray analysis of CA3 gene expression

The induction protocol used here was previously demonstrated to produce a robust signal in the CA3 region of the hippocampus in young adult rats [18]. In the current experiment, CA3 mRNA, from tissue dissected immediately after probe trial completion (approximately 2 hours after task onset) was analyzed on Affymetrix Rat 230-2.0 arrays for all AU and AI subjects, with each array measuring mRNA levels from a single subject. Rat 230-2.0 arrays include more than 31,000 probesets to interrogate approximately 28,000 genes. Data were analyzed using a standardized procedure that includes quality control analysis, gcRMA normalization and a low expression cut-off employed to remove genes with extremely low or no expression (see methods for details). Quality control analysis identified one outlier array, which was removed for all subsequent analyses. Multi-dimensional scaling (MDS) and Principle Component Analysis (PCA) are unsupervised analysis tools that assess broad gene expression patterns across arrays. Data from these analyses can be represented in two-dimensional plots in which each dot represents a single array/subject and arrays with more similar expression patterns are located closer in proximity. 

**Figure 2:**
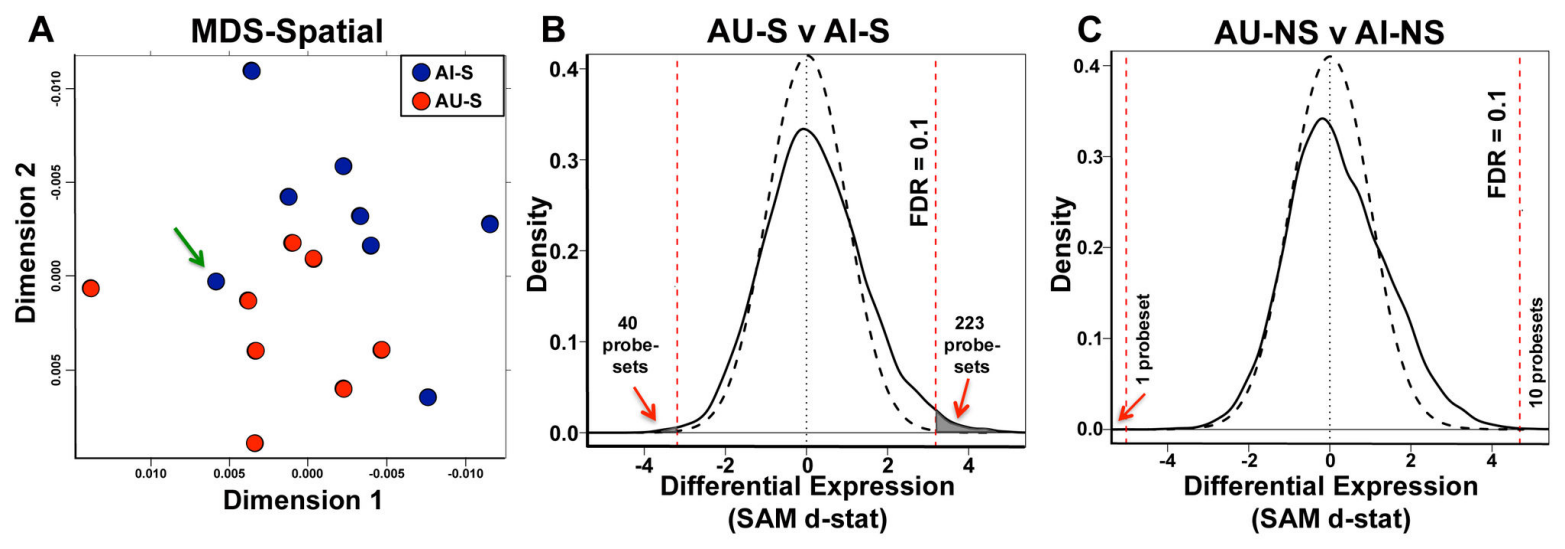
Spatial training induces differential AU and AI expression profiles. **A**. CA3 gene expression MDS (distance = 1 – r) analysis of AU and AI spatially trained rats shows clustering of AU-S subjects. Each point within the graph represents the array for a single subject colored by cognitive phenotype, as indicated. The distance between points is an indication of relative similarity of mRNA profiles. AU arrays (red) tend to cluster together, at least partially segregated from AI arrays (blue). Green arrow points to an AI rat with borderline LI (LI=254); LI range for AU is 240 or lower. **B**. SAM d-statistic density plot comparing AU-S expression to AI-S, shows large numbers of genes differentially expressed between groups. The dashed black line represents the expected, random d-statistic density distribution while the black solid curve represents the distribution observed AU-S/AI-S comparison. Numbers of genes differentially expressed at an FDR = 0.1 (depicted by red dashed lines) are indicated on each graph. The gray colored area under solid curve represents the numbers of probesets that meet an FDR of 0.1. Positive SAM d-stat values indicate increased gene expression in AU-S with most of the differentially expressed genes increased in AU. **C**. SAM d-statistic density plot comparing AU-NS expression to AI-NS, while similar in shape to B., shows few genes differentially expressed between groups. The dashed black line represents the expected, random d-statistic density distribution while the black solid curve represents the distribution observed AU-NS/AI-NS comparison. Numbers of genes differentially expressed at an FDR = 0.1 (depicted by red dashed lines) are indicated on each graph. Positive SAM d-stat values indicate increased gene expression in AU-NS.

Using the behavioral performance as a guide to direct gene expression comparisons, we first looked for differences between the expression profiles of AU and AI rats trained in the spatial condition of the induction protocol prior to sacrifice (AU-S v AI-S). Because the AU-S subjects showed clear evidence of performance based on spatial information while the AI-S did not, we expected learning induced expression to differentiate these two subject groups. Global analysis by MDS and PCA indicated that mRNA profiles largely grouped the arrays by cognitive phenotype (Figure 2A and Figure S1A) with AU-S subjects predominantly clustered together. One AI-S subject appeared to cluster with AU-S arrays (arrow) and subsequent examination found this subject to have a LI score (254) near the cut-off between AU and AI (LI=240). Significance Analysis in Microarray (SAM), which utilizes planned comparisons to determine how many and which genes exhibit differential expression between the groups, found over 250 probesets differentially expressed between AU-S and AI-S rats using stringent criteria [Figure 2B; false detection rate (FDR) of 0.1; gray shaded areas represent differentially expressed genes], with the vast majority of those genes increased in AU-S subjects relative to AI-S, consistent with the expectation of learning induced gene expression in AU rats. 

We performed an analogous comparison between AU and AI rats in the control task. As expected these two groups did not differ behaviorally in task performance and had limited opportunity for learning induced gene expression explicitly tied to an escape location in the task. However both groups had an equivalent opportunity for physical activity-induced gene expression during exposure to a novel environment. The AU-NS v AI-NS SAM comparison exhibited a similarly shaped distribution as the spatial comparison (Figure 2C), but far fewer probesets met the FDR cutoff suggesting less robust differences between these two groups. Thus, the microarrays for the CA3 region distinguished AU from AI rats in the spatial condition in detecting large numbers of differentially expressed genes, with a less prominent difference detected in the non-spatial condition. 

**Figure 3:**
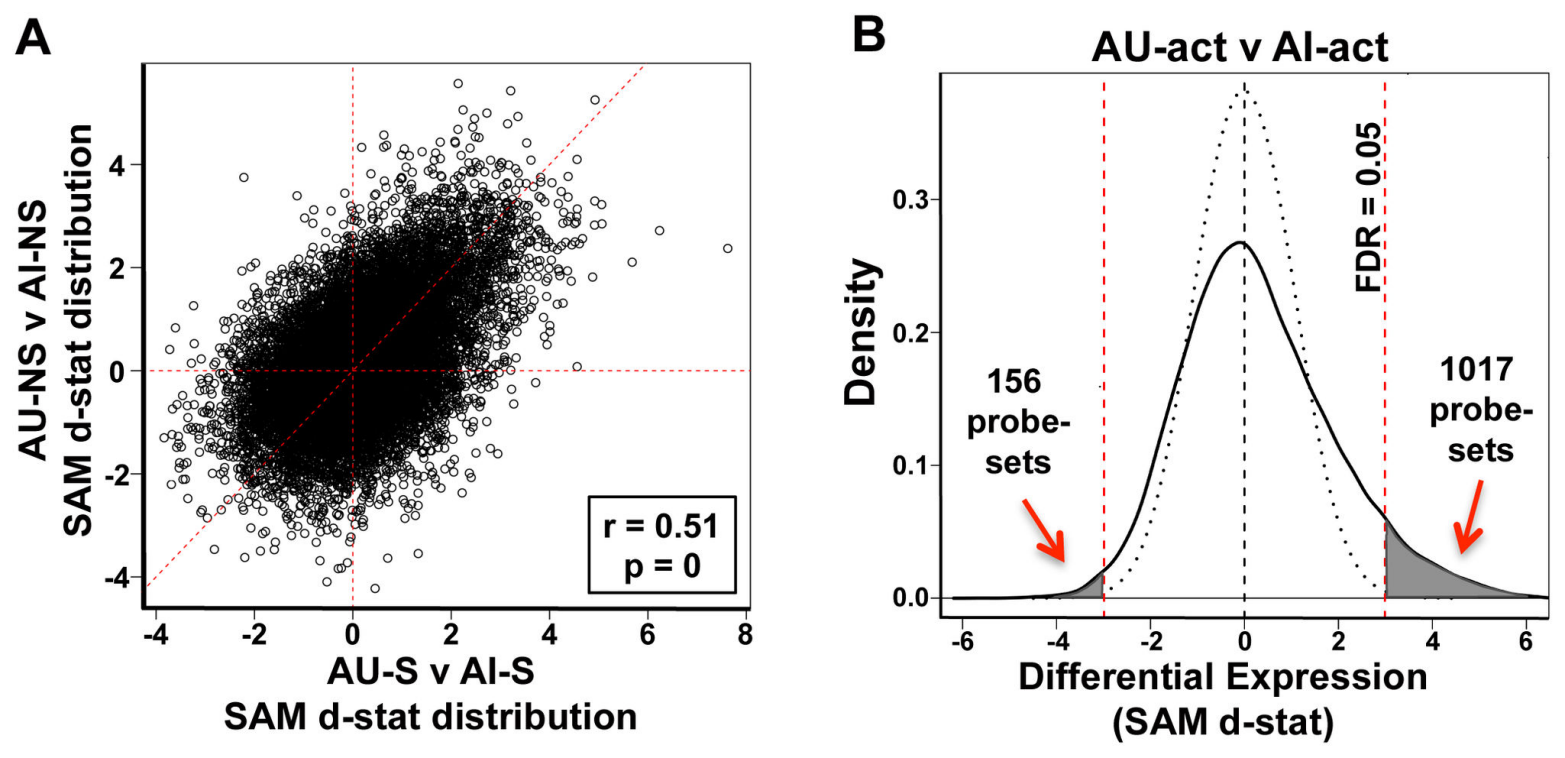
CA3 behaviorally activated profiles distinguish AU from AI rats. **A**. Scatter plot of SAM d-statistic values for the AU-NS to AI-NS comparison (X-axis) against those of the AU-S to AI-S comparison (Y-axis) shows a strong correlation (r=0.51, p=0) between S and NS profiles. Each circle represents a single probeset (N>15,000 probesets). **B**. SAM d-statistic density plots comparing all behaviorally activated AU (AU-S + AU-NS) to all behaviorally activated AI subjects (AI-S + AI-NS) show large numbers of genes differentially expressed between groups. Combined groups are designated AU-act and AI-act respectively. The dotted black line represents the expected, random d-statistic density distribution while the solid back curve represents the distribution observed in the comparison between AU-act and AI-act. The gray colored area under solid curve represents the probesets that meet an FDR of 0.05 (red dotted lines). Positive d-statistic values represent increased expression in AU-act relative to AI-act. Most of the genes were increased in AU-act as indicated, with numbers of differentially expressed probesets indicated on the graph.

In addition to providing an opportunity to differentiate AU and AI gene expression patterns, the induction protocol allowed for the distinction between gene expression induced by spatial task-relevant information and that induced by exposure to the novel environment with minimal relevance of spatial information for task performance. Therefore, we asked if the S and NS induction conditions produced different gene expression profiles within each of the AI and AU groups. Because the AI rats were impaired in learning the platform location, showing no spatial bias in either S or NS condition, no difference was expected in the expression profiles between AI-S and AI-NS subgroups. As anticipated, neither MDS analysis nor SAM analysis (Figure S1B, C) found any significant differences between spatial and non-spatial training conditions for AI rats. Surprisingly, however, the expression patterns for AU rats between S and NS training conditions also did not differ significantly, despite clear evidence of behavioral learning as a function of the S induction protocol (Figure S1D, E). Thus within a cognitive group, S and NS conditions produced quite similar profiles in both AI and AU subjects. To further explore the relationship between expression induced by the two task conditions, we asked if AU/AI differences found in the S condition were similar to the AU/AI differences in the NS condition. To test this, we plotted d-statistic values for the AU-S v AI-S comparison against the d-statistics of AU-NS v AI-NS comparison for each probeset (Figure 3A). These d-statistic distributions were highly and significantly correlated (r=0.51; p=0) indicating that cognition-dependent group differences were quite similar, regardless of the induction condition. 

### Combined spatial and non-spatial profiles exhibit features similar to spatial alone

The correlation across S and NS conditions suggests similar gene expression regulation independent of spatial information relevant to the task contingencies that differed across the two induction protocols. In light of the similarity between S and NS profiles and the increased power gained with a larger sample size, we combined subjects from both training conditions into a single group for AU (AU-S + AU-NS) and AI (AI-S +AI-NS) subjects and then compared those groups to discover differentially activated expression patterns related to cognitive phenotype. With the inclusion of the NS condition, these profiles can no longer be considered ‘learning activated’ with respect to the experimentally controlled variables of platform location and extramaze cues but reflect experience dependent profiles, which we will hereafter refer to as ‘behaviorally activated’ (groups denoted AU-act and AI-act). Similar to the data restricted to the spatial training condition (shown in Figure 2A), the MDS plot of all aged rats that received behavioral induction training (AU-act and AI-act), color-coded by cognitive status, shows a clustering by aged phenotype, with AU-act arrays somewhat segregated, although not completely, from AI-act subjects (Figure S2). Consistent with those data, SAM found more than 1000 probesets differentially expressed between AU-act and AI-act rats at a FDR of 0.05 (Figure 3B, gray shaded areas). In further agreement with the analysis that considered only rats in the spatial training condition, the vast majority of differentially expressed genes were increased in AU-act relative to AI-act rats. Because behavioral performance for the AU-act and AI-act groups did not differ with respect to physical activity (e.g. escape latency) either within- or across induction protocols, differences in behaviorally activated expression between those groups could more generally reflect information processing engaged during task performance by a novel environment. 

To characterize those genes differentially expressed between AU-act and AI-act profiles, we performed functional groups analysis using the free functional annotation tool, DAVID, which examines gene lists for over-representation of genes belonging to a particular functional group as determined by gene ontology, KEGG and other functional annotation databases [21,22]. Using the list of genes that showed increased expression in AU-act relative to AI-act (FDR<0.05), we identified 105 functional groups that met a Bonferroni corrected p<0.05 (Table S1). Analysis of functional groups revealed increased expression of genes involved in nucleotide (ATP) binding, vesicle/protein transport, cytoplasmic/synaptic vesicles, GTP binding, synapse, and synaptic transmission: all groups involved in synaptic plasticity and learning. Functional annotation of those genes with decreased expression (FDR<0.05) in AU-act rats found no groups that met a Bonferroni corrected p<0.05, although 28 groups were found that met an uncorrected p<0.05. Those gene groups were quite different from the increased gene groups and were ones involved in programmed cell death and signal transduction. 

### Basal expression patterns differ from behaviorally induced expression

**Figure 4:**
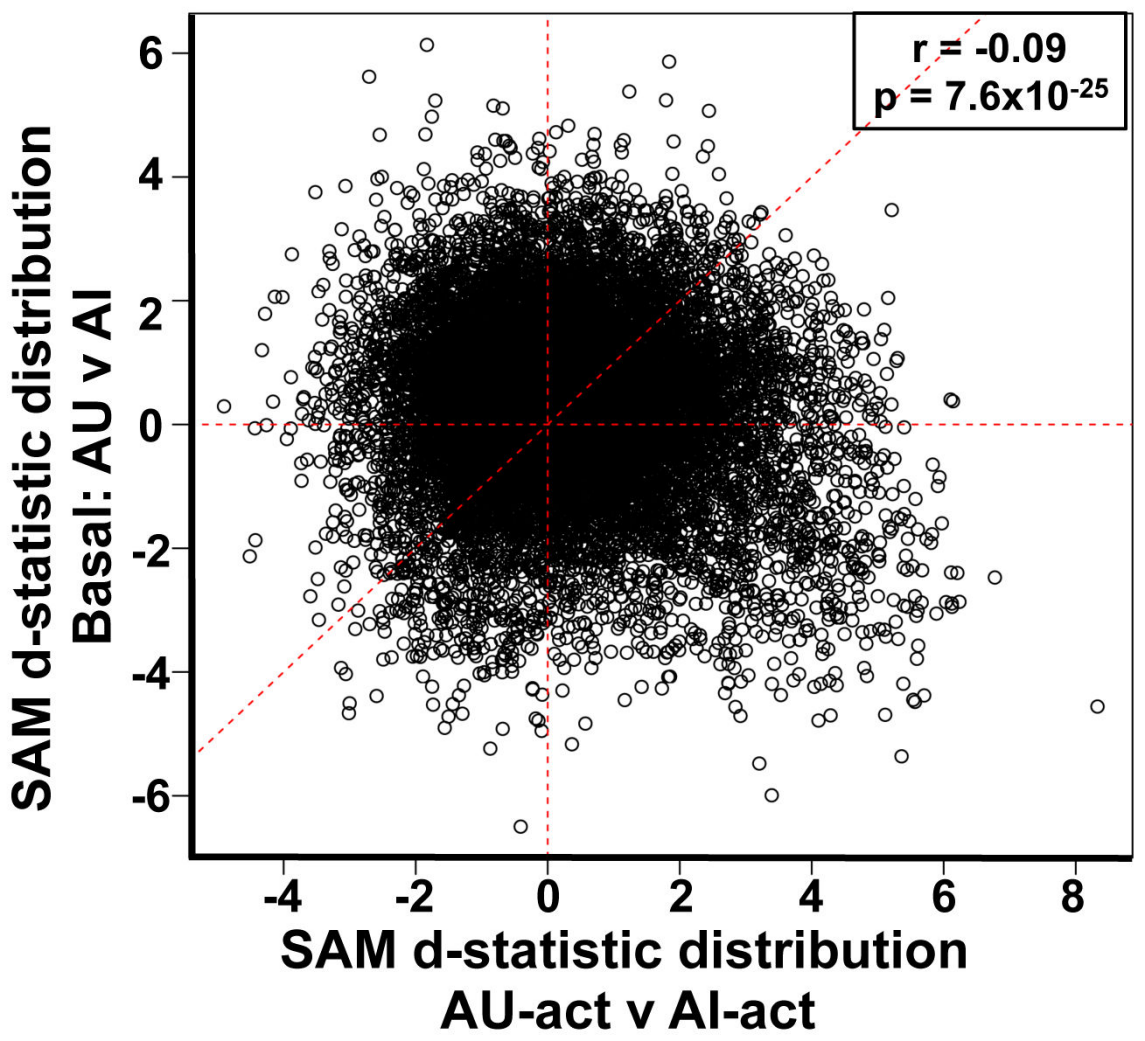
Behaviorally activated expression profiles are distinct from basal expression. SAM d-statistic values derived from basal CA3 gene expression comparison between AU and AI rats [14] were plotted against AU-act v AI-act SAM d-statistic values for each probeset. Each dot represents a single probeset (N>15,000 probesets). Correlation r-value and accompanying p-value are indicated on the graph. A slight negative correlation indicates negligible similarity between the basal and activated expression profiles.

The large number of increased genes in AU-act subjects relative to AI-act supports the notion that the AU arrays represent behaviorally-induced profiles. We found further evidence of this interpretation by comparing the current arrays with our published microarray dataset under basal conditions, when aged rats were sacrificed directly from the home cage [14]. Table 1 summarizes and compares experimental designs across microarray datasets. It is important to note that with respect to the reliability of basal expression findings, critical expression differences associated with the aged phenotypes from the basal microarray were replicated with independent sets of AU and AI subjects and distinct methods for evaluating gene expression levels. Because the basal arrays were performed as a separate experiment, albeit using identical procedures used here, we did not directly compare probesest intensities across experiments. Instead, we compared the relative differences between AU and AI rats in each dataset. Specifically, we plotted SAM d-statistics derived from basal AU v AI comparisons against SAM d-statistics derived from AU-act v AI-act for each probeset (Figure 4). A positive correlation would indicate similarities in the direction and magnitude of gene expression differences between subject groups in the basal condition and after behavioral activation. Instead, the plot exhibited a slight negative correlation that was significant due to the large number of datapoints (>15,000 probesets). The absence of any positive correlation strongly suggests that the basal profiles differed significantly from the activated datasets. Significantly, the same pattern was found when comparing SAM d-statistics of the S condition (Figure S3A) or the NS condition (Figure S3B) alone to the basal array. 

**Table 1:** Microarray dataset descriptions.

**Dataset**	**Platform**	**Subjects**	**Standardized Behavioral Characterization**	**Induction Protocol**	**Publication**
**Aged Behaviorally Activated**	Affymetrix	Aged Unimpaired	Yes	Yes	N/A
	Rat 230-2	Aged Impaired			
	(~31K probesets)				
**Basal Aged**	Affymetrix	Aged Unimpaired	Yes	No	Ref 14
	Rat 230-2	Aged Impaired			
	(~31K probesets)	Young			
**Young Spatial Learning**	Affymetrix	Young	Yes	Yes	Ref 18
	Rat 230A				
	(~15K probsets: subset of 230-2)				

### Learning genes in behaviorally-activated aged rats

**Figure 5:**
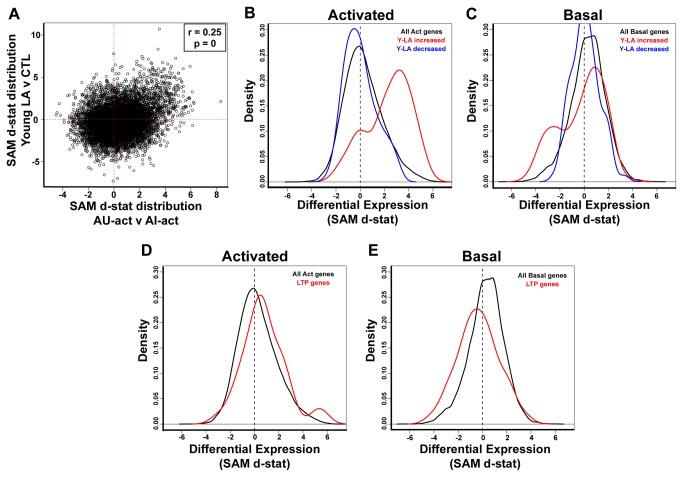
Learning genes are increased in AU relative to AI with behavioral experience. **A**. SAM d-statistic values derived from young learning (LA) v control (CTL) [18] were plotted against AU-act v AI-act SAM d-statistic values for each probeset. Each dot represents a single probeset (N>7,000 probesets). The positive correlation indicates significant similarity between young learning and aged activated expression profiles. **B**. CA3 genes that were significantly modulated by a learning episode in young rats [18] were tested for differential expression between AU-act and AI-act rats from the current study. Positive d-stat values indicate increased expression in AU-act subjects. Genes observed to increase expression with learning in young rats (red line) were also significantly increased in AU-act compared to AI-act subjects (N=189; p=7.7 x10^-44^). Black line represents the distribution of all genes. **C**. Similar to B., learning-activated genes in young rats were examined with respect to differences in AU and AI rats under basal conditions (red line), revealing a significant decrease in AU rats compared to AI (N=173; p=0.0067). In B and C, genes observed to decrease expression with learning in young rats (blue line) did not have a significantly different distribution in aged rats with behavioral activation (blue line shown in B, N=81; p=0.12), although the distribution was marginally decreased in AU relative to AI rats under basal conditions (blue line shown in C, N=81; p=0.067). **D**., **E**. Genes known to be increased with LTP [18] were also tested for differential expression between AU and AI rats in (**D**) activated and (**E**) basal microarray datasets. Similar to learning induced genes, LTP genes (red line) were significantly increased in AU-act rats (N=54; p=0.03) and significantly decreased in AU rats under basal conditions (N=44; p=0.0056). The black line in panels B and D is equivalent to the solid black line in Figure 3B.

While the data above suggest that AU rats recruit a generally similar CA3 gene expression profile during performance in a novel environment, the behavioral search in the probe trial indicates that the AU-act expression profile occurs in the context of using newly acquired spatial information. To further examine that interpretation, we asked if the genes differentially expressed between AU-act and AI-act correspond to genes known to change with spatial learning in young animals. To address this question, we leveraged our previous microarray study performed in young rats that examined spatial learning induced gene changes in the CA3 hippocampal subfield (Table 1) using the same induction protocols [18]. To assess overlap with the behaviorally-activated dataset in the current study, we first plotted SAM d-statistic density distributions of the young spatial learning condition (LA) v the young control (non-spatial, CTL) group against the AU-act v AI-act SAM distribution observed in the current study. Indeed, as shown in Figure 5A, there was a very significant positive correlation indicating that those genes differentially expressed after behavioral activation in aged rats showed similar expression differences with learning in young rats. To further investigate the origins of this correlation, we examined lists of probesets either uniquely increased or decreased by spatial learning in young rats (see [18] for details and gene lists). We examined the SAM d-statistic distribution of the members of those lists compared to the distribution of all genes in the AU-act v AI-act comparison. The d-statistic distribution of probesets which had been found to increase with spatial learning in young rats showed a significant positive skew in AU-act rats relative to AI-act subjects (Figure 5B, red line), confirming that many learning-activated genes found in young rats were also up-regulated in AU rats, relative to AI, by the behavioral induction protocol. A direct comparison of gene lists revealed that 44% of probesets significantly increased with spatial learning in young rats were also significantly up-regulated in AU-act relative to AI-act rats at an FDR ≤ 0.05 (92 probesets; Table S2). In contrast, the distribution of down-regulated genes observed in conjunction with spatial learning in young rats was not different from the distribution of all genes in the AU-act v AI-act comparison (Figure 5B, blue line). Thus the positive correlation found in Figure 5A is driven largely by genes that are also increased with spatial learning in young rats. These data confirm that many genes with elevated expression in AU rats relative to AI after behavioral induction are likely relevant to spatial learning, playing a role in the successful performance of AU rats in spatial memory tasks. 

The d-statistic distribution of the increased and decreased gene sets for spatial learning in young rats was also examined in our basal aged rat dataset (Figure 5C). Interestingly, those genes with increased expression as a function of spatial learning in young rats exhibited a significant negative shift in the basal AU v AI SAM comparison, indicative of lower expression in AU rats compared to AI rats, suggesting that at least a subset of learning-related genes that were increased in young rats actually showed reduced expression in AU vs AI rats under basal conditions. Those genes with decreased expression as a function of spatial learning in young rats also showed a slight negative skew of marginal significance. Considered together, these data demonstrate that with behavioral induction, AU rats exhibit a profile of gene expression, relative to AI rats, that resembles the expression profile observed with learning-activation in young rats and that some of those same genes appear to have reduced basal expression in AU relative to AI rats. 

In a manner similar to our prior analysis of spatial learning induced genes in young rats, we examined a set of genes known to be specifically activated as a function of long-term potentiation (LTP) [18]. As expected this gene set also showed a significantly different distribution comparing AU-act to AI-act rats (Figure 5D), with a skew clearly evident towards increased expression in AU-act rats. That differential distribution appears to be driven by several genes with highly significant d-statistics including APP, Mapk1, Ntrk2, Snap25 and Stx1b. Consistent with behavioral induction of expression, those genes showed either no expression difference between AU and AI in the basal arrays or an AU decrease relative to AI under basal conditions. Indeed overall, the LTP genes had a significant negative skew, indicative of lower expression, in AU rats relative to AI under basal conditions (Figure 5E). 

**Figure 6:**
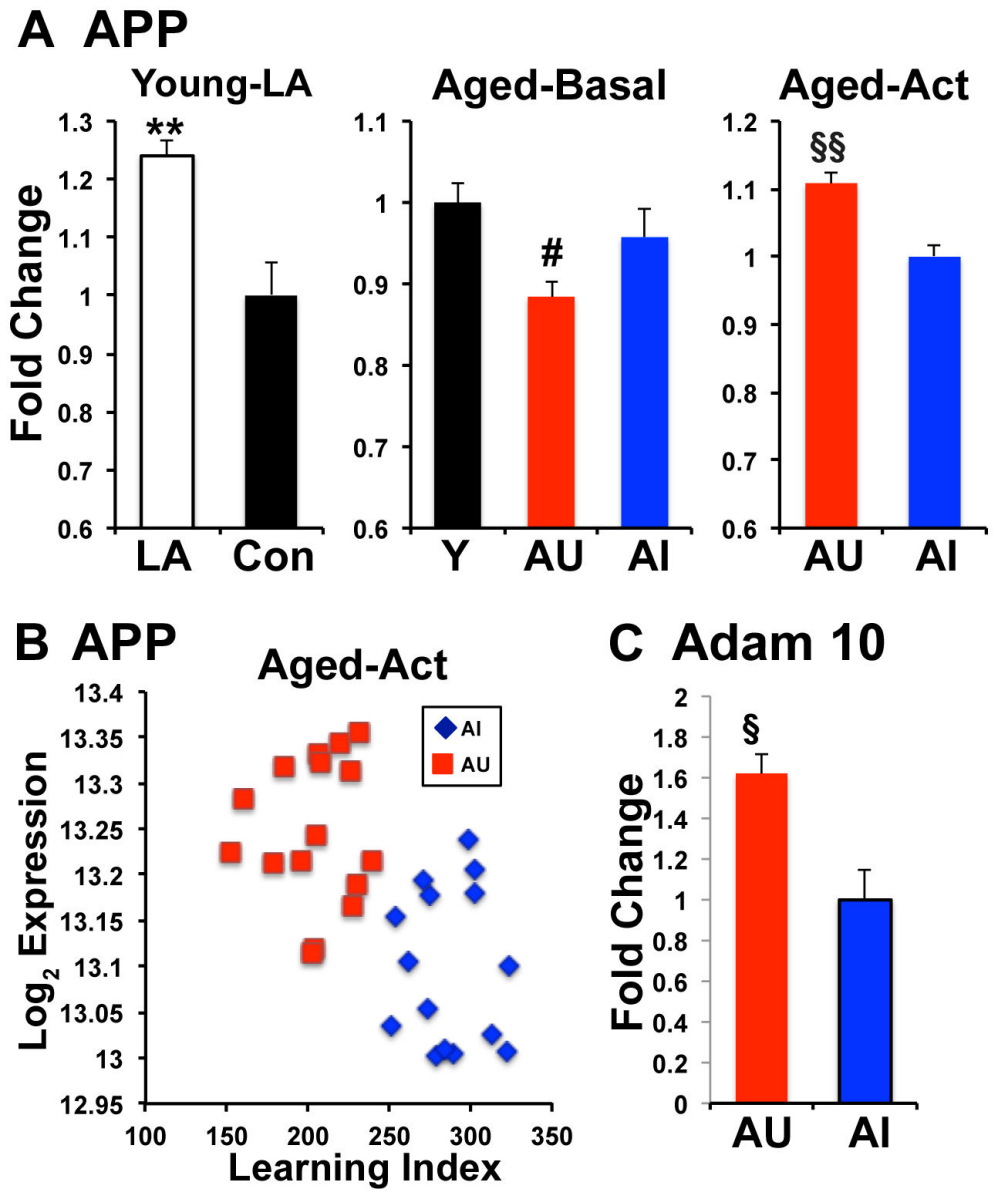
APP gene illustrates expression changes across conditions. **A**. The Alzheimer’s disease gene, App (amyloid precursor protein) has decreased expression in AU relative to AI and Y under basal conditions. App also exhibited increased expression in Y rats in the spatial learning-activated protocol. Behaviorally activated expression in AU is significantly higher than AI. **B**. Individual subject expression levels (log_2_) for App from the Aged activated dataset are illustrated in the scatter plot. **C**. Adam10, an App α-secretase gene shows a concomitant increase in expression in AU-activated rats. Error bars represent SEM. **, p<0.002 v Con; #, p<0.005 v AI; §§, p< 0.00005 vs AI-Act; §, p<0.0.005 v AI-Act.

To better illustrate the differences in expression patterns across the three datasets, Figure 6A and B shows expression levels for the amyloid precursor protein gene (App) gene that was activated with spatial learning in young rats and showed a behaviorally induced increase in AU-act rats relative to AI-act. Of particular relevance to cognitive aging because of its association with Alzheimer’s disease (AD) pathology, App also exhibited reduced expression in AU rats relative to both young and AI in the basal gene array dataset. These data are consistent with an interpretation that App is activated with behavior in AU rats relative to the activation in AI rats, but maintained at reduced expression levels under basal conditions, indicating tighter control in a homecage environment in aged rats with preserved cognitive function. Interestingly, concomitant with the increased App expression in AU-act rats, the α-secretase, Adam10 is also upregulated in AU-act relative to AI-act rats (Figure 6C). Cleavage of App by Adam10 biases production towards the non-amyloidogenic sAppα and away from Aβ, the toxic peptide that accumulates in AD [23].

### AU specific genes downregulated under basal conditions are differentially regulated after behavioral induction

**Figure 7:**
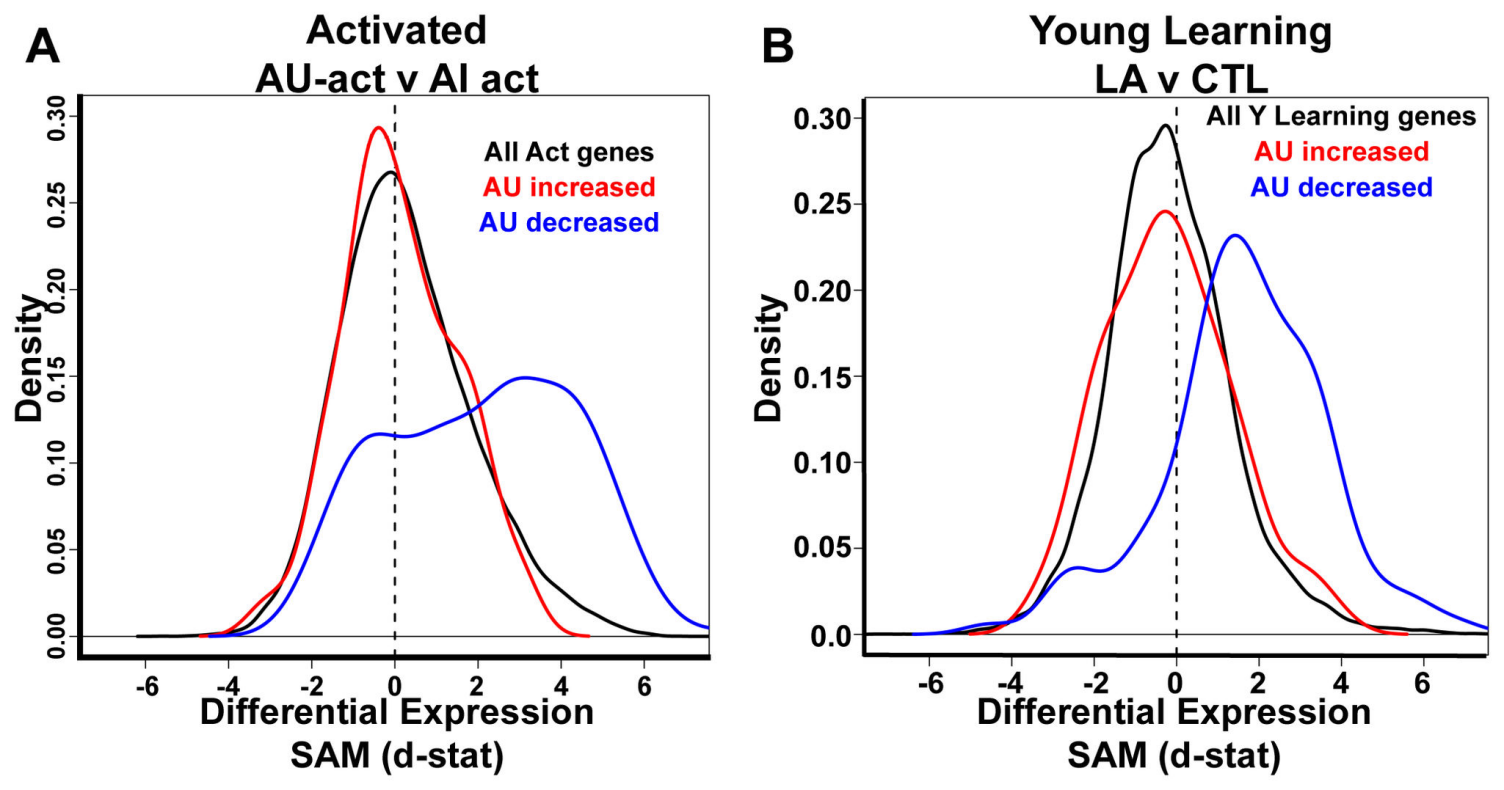
AU specific genes shift expression with induction protocol and learning. **A**. CA3 AU specific genes, derived from the basal aging dataset [14], were tested for differential expression in the behaviorally activated dataset (AU-act v AI-act). Previously identified AU genes that were selectively decreased under homecage conditions (N=169; blue line) were significantly increased in AU-act rats (p=2 x10^-25^), whereas the genes that had elevated expression in AU rats under homecage, basal conditions (N=117; red line) were not significantly different from the distribution of all genes (black line) in the activated dataset (p=0.42). Positive d-stat values indicated increased expression in AU subjects relative to AI. The black line (representing all genes) in panel A is equivalent to the solid black line in Figure 3B. **B**. CA3 AU specific genes, derived from the basal aging dataset [14], were also tested for differential expression in the learning-activated dataset for Y rats. AU decreased genes (N=113) were significantly increased in Y rats (learning activated vs control protocols, blue line compared to all genes represented by the black line, p=1.3 x10^-25^) while the AU genes with increased expression under homecage basal conditions (N=82) were not significantly different from the distribution of all genes (red line compared to black line; p=0.8). Positive d-stat values indicated increased expression with spatial learning. The black line represents the distribution of all genes in the learning v control comparison in young rats in the arrays [18].

The cross dataset comparisons described above suggest that AU rats exert distinctive control over the basal expression of genes, which become regulated in the opposite manner by behavioral-activation in the AU profile and thus give rise to the slight negative correlation noted in Figure 4 and Figure S3. To directly test this idea we returned to our analysis of the basal aging dataset in which we had defined a list of genes that were uniquely upregulated or downregulated in AU relative to both young and AI rats [14]. Because these genes distinguished AU rats from both young and AI rats, they represented potential mechanistic features that support intact cognitive capacity in the aged brain. For membership in this list we required that genes show significantly different expression (p<0.05) from both Y and AI in the same direction and that Y and AI not show significantly different expression (p>0.3). These criteria generated a list of 172 AU-specific decreased probsets and 117 AU-specific increased probesets [14]. We examined the distribution of these probeset lists in the AU-act v AI-act comparison (Figure 7A) and found that while the basal AU-specific genes with increased expression did not differ significantly from all genes in the aged behaviorally-activated dataset, the decreased AU genes showed a decisive positive shift, indicative of increased expression in the AU-act rats. These data are alternatively illustrated by color-coding the scatterplot from Figure 4 (basal aging vs aged behaviorally activated SAM d-statistic comparison) with basal AU-specific increased genes in red and basal AU-specific decreased genes in blue (Figure S4A). Only the blue dots show a greater preponderance to the right of the vertical red zero line indicating increased expression in AU-act rats. Remarkably, more than 50% of basal AU decreased genes were significantly increased (at p<0.05; 91 probesets) in AU-act relative to AI-act subjects (37% at FDR<0.05). The DAVID functional group analysis of these genes is also consistent with the overall AU-act gene groups, including many involved in vesicle transport and neurotransmission (genes from ‘GO: Synaptic transmission’ functional group illustrated in Figure S5). These data suggest that AU rats exert tight control of expression under basal conditions of genes involved in neurotransmission, which can then be up-regulated in a behaviorally activated state, as observed with the behavioral-induction protocol. Consistent with the interpretation that the basal list for selective AU decreased expression contains behaviorally relevant genes for learning, the distribution of these genes in the young spatial learning dataset also shows a significant positive shift indicative of genes recruited during learning (Figure 7B and Figure S4B). 

### Increased inhibition of is a signature of AU-act rats

**Figure 8:**
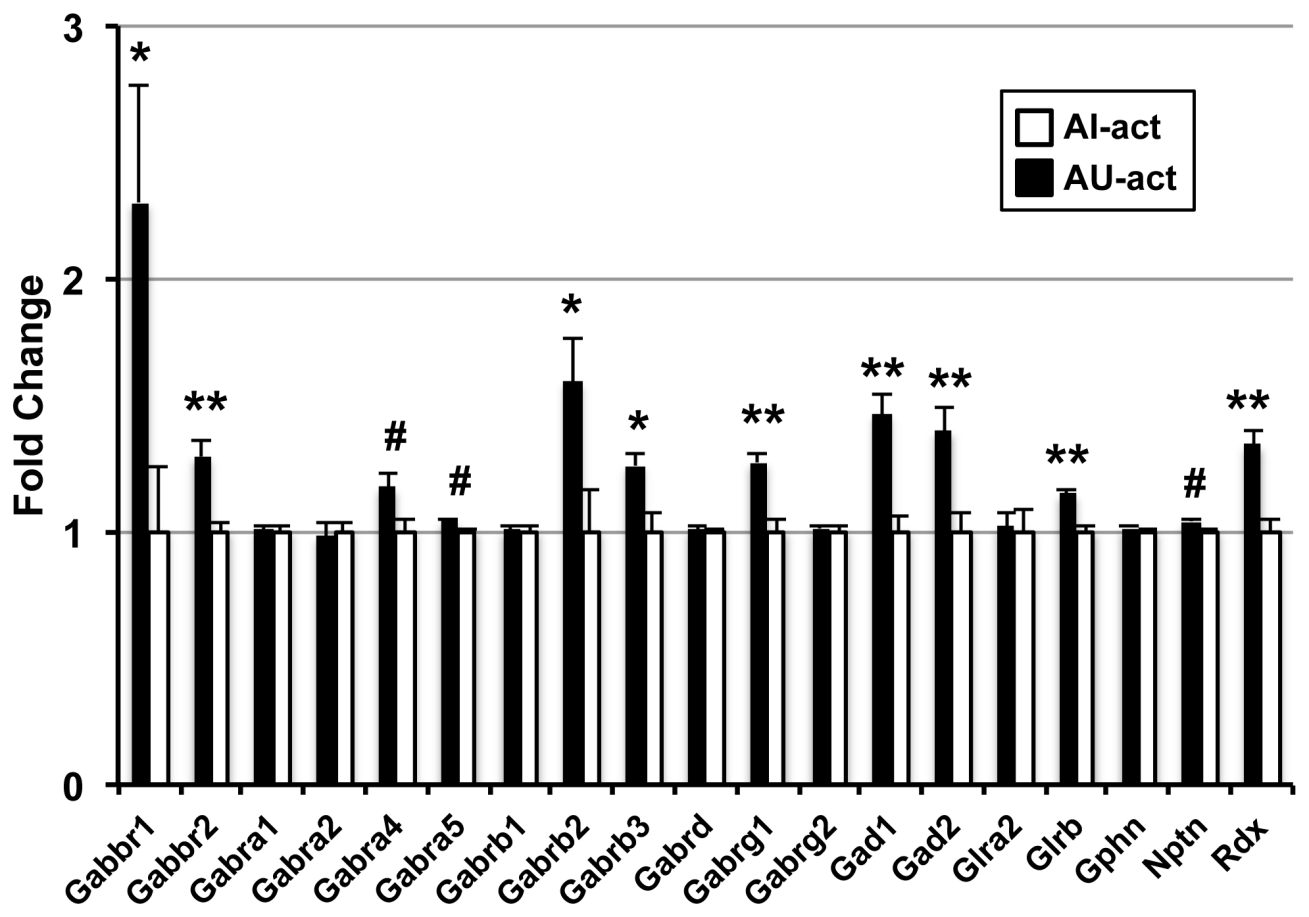
Genes associated with inhibitory neural function are increased in AU-act subjects. Relative microarray intensities of genes encoding inhibitory synaptic/extrasynaptic receptors and synthetic enzymes are plotted for AU and AI activated rats. **, p<0.05 and FDR<0.05; *, p<0.05 and FDR<0.1; #, p<0.05 and FDR<0.15. Error bars indicate SEM. Abbreviations: Gabbr1, GABA-B receptor 1; Gabbr2, GABA-B receptor 2; Gabra1, GABA-A receptor α1; Gabra2, GABA-A receptor α2; Gabra4, GABA-A receptor α4; Gabra5, GABA-A receptor α5; Gabrb1, GABA-A receptor β1; Gabrb2, GABA-A receptor β2; Gabrb3, GABA-A receptor β3; Gabrd, GABA-A receptor δ; Gabrg1, GABA-A receptor γ1; Gabrg2, GABA-A receptor γ2; Gad1, glutamate decarboxylase 1; Gad2, glutamate decarboxylase 2; Glra2, glycine receptor, a2; Glrb, glycine receptor, b; Gphn, gephyrin; Nptn, neuroplastin; Rdx, radixin.

One feature of cognitively impaired individuals in both humans and rodent populations is the presence of CA3/DG overactivity. Thus, it was particularly striking to observe increased expression for a series of molecules involved in inhibitory neuronal function, which could be relevant for the control of CA3 excitability in AU-act rats. Figure 8 shows relative microarray expression levels of all GABA and Glycine receptors, as well as both GABA synthetic enzymes and three GABA/Glycine receptor-specific anchor proteins gephyrin, neuroplastin and radixin that exceeded the low expression cut-off in the behaviorally-activated dataset. Nine of the 19 genes showed significantly increased expression in AU-act rats at an FDR<0.1 with three more genes having a p<0.05 with slightly higher FDRs. We previously independently confirmed expression differences in Gabra5, which showed differential expression in our basal microarray study, but most of these genes associated with neuronal inhibitory function show little differential expression under basal conditions [14]. A subset of these genes also showed learning induced increased expression in young rats, but the magnitude and extent was unique to the AU-act dataset. Because our neural recording studies have shown that CA3 neurons in AI rats have elevated firing rates and fail to rapidly encode new information in a manner comparable to young adults [6,8], this AU-act profile may significantly contribute to the unimpaired aged phenotype supporting enhanced inhibitory control. This inhibition may be particularly important for proper CA3 function because the CA3 recurrent collaterals provide a feed-forward loop for excitatory neurotransmission, providing the majority of excitatory drive onto CA3 pyramidal neurons. Increased expression of genes associated with inhibitory function may act as a brake on CA3 excitation in AU rats, contributing to the ability of those rats to regulate excess neuronal activity and maintain the ability to rapidly encode new information in the network. 

## Discussion

Our previous research has directed attention to the CA3 subfield of the hippocampus as a focal point for neurobiological alterations underlying age-related memory decline [12,14,24]. Indeed, insights gained from this model, at molecular and systems levels of analysis, have recently allowed for a translation to elderly humans and patients with amnestic mild cognitive impairment using functional magnetic resonance imaging (fMRI) [9,11,25]. Those clinical investigations were built primarily on the basis for CA3 dysfunction identified in aged rats with memory impairment. Here we compare behaviorally activated CA3 gene expression profiles of rats that age without cognitive decline (AU) to cohorts with significant impairment (AI) to investigate neurobiological features that may contribute to preserved cognitive function in aged individuals. Our results suggest that while AU rats engage some of the same molecular pathways as young rats performing a spatial memory task, significant differences in gene regulation are evident between young and AU subjects. Some unique AU expression patterns suggest engagement of mechanisms designed to control excitability both under basal and activated conditions.

We used gene expression microarrays to generate the profiles of the aged rat CA3 transcriptome after behavioral activation. We also leveraged our previously published CA3 microarray datasets to provide comparison profiles for basal gene expression and for learning-induced expression in young subjects. In the current study, behaviorally activated gene profiles distinguished aged unimpaired rats from aged impaired, consistent with our finding of differential basal CA3 profiles for those aged subgroups [14]. However, across the spectrum of aged cognitive performance, the overall mRNA profiles after behavioral activation showed no positive correlation with the basal profiles, indicating further distinctions in gene expression differentiating AU and AI subpopulations under basal and behaviorally-activated conditions. 

Genes with increased expression clearly predominated over genes with decreased expression in AU rats relative to AI rats after behavioral activation. Further analysis revealed that genes in the AU-act profile with increased mRNA expression belonged to functional groups involved in neurotransmission and synaptic plasticity. The proposed contribution of such activated AU gene expression to the acquisition of new information in the behavioral task is consistent with the substantial overlap in AU-act gene expression with genes increased in CA3 after spatial learning in young adult rats [18]. At the same time, the data suggest that gene expression was recruited somewhat less selectively in AU rats compared with young adults under the conditions of the two protocols used for gene induction. AU rats did not exhibit a significantly different CA3 expression pattern between the S and NS versions of the induction protocol but rather showed a relatively similar transcriptional response across those conditions. In contrast, our previous experiment demonstrated that young rats produced a more distinctive CA3 gene expression profile between the S and NS induction protocols ([18]; S and NS were called LA and CTL respectively in this paper). Nonetheless, AU rats clearly used spatial information to support behavioral learning as manifest in a strong spatial bias in probe trial performance searching at the position of the platform that defined the escape location during training in the S-protocol. The ability of the AU rats to encode spatial information in the absence of a gene expression S-NS difference could reflect a mechanistic shift, possibly to post-transcriptional/translational mechanisms, that facilitates learning in the environment of the aging brain. 

Consistent with published studies demonstrating increased gene expression after watermaze training, including visible platform training, relative to naïve controls [16,26], comparisons between the profiles in basal and behavioral induction conditions suggest that AU rats exhibit robust activation of mRNA relative to AI rats in response to performance of a behavioral task in a novel environment. Thus, in comparison, AI rats are either unable to activate gene expression, or do so less robustly. This interpretation is supported by studies in this model of the functional integrity of synaptic plasticity mechanisms, which are consistent with differential AU/AI profiles under basal and activated conditions. Examination of long-term potentiation (LTP) and long–term depression (LTD) in hippocampal slice preparations have found subfield specific LTP and LTD deficits in AI rats with relative integrity of these mechanisms in AU subjects [27-29]. Due to impaired plasticity, AI rats would be expected to have a reduced ability to activate downstream signaling pathways and to appropriately regulate gene transcription in response to new information. Whereas preserved plasticity in AU rats provides an intact pathway for gene induction and production of the molecular substrates of new information encoding and consolidation. 

In our original assessment of basal aging microarrays for the AI and AU phenotypes, we identified a set of genes that were uniquely differentially expressed in AU rats relative to both young and AI [14]. Such genes represent an AU signature that could elucidate mechanisms engaged by AU individuals to maintain function in an aging context. We examined the distribution of those genes in the behaviorally activated condition and found that remarkably, almost half of the genes that were significantly repressed in AU rats, under basal conditions, were among those increased in behaviorally activated AU rats relative to AI. Because many of those genes are involved in neurotransmission by functional group analysis, those results suggest tighter control over neuronal activity in homecage basal conditions in the aged rats with preserved cognitive abilities. The distinctive regulation of those genes might contribute to control over neuronal excitability, a critical aspect of aged individuals with intact cognitive function. 

The pattern of gene regulation noted above was illustrated by the amyloid precursor protein (App) gene (Figure 6), which shows repression in basal AU and significantly greater expression with behavioral activation in AU-act relative to AI-act. App expression was also similarly increased in young animals as a consequence of spatial learning [18]. App is a multifunctional protein central to a pathophysiological pathway in Alzheimer’s disease (AD). At the same time, multiple cleavage products have been reported to have differential activities with sAppα, an extracellular non-amyloidogenic fragment, demonstrating neuroprotective effects and contributing positively to both short and long term memory [23]. The Aβ fragment, believed to be critical in the pathophysiology of AD, is neurotoxic upon accumulation, but modulates synaptic plasticity, perhaps as a negative feedback mechanism contributing to homeostatic maintenance of synaptic plasticity [30]. Generation of these two products is mutually exclusive based on the cleavage site of sAppα which lies in the middle of the Aβ fragment. Within that framework, the differential regulation of App mRNA levels in AU rats might be part of a program to regulate the differential effects of the various App cleavage products. Decreased expression at baseline could reduce excess production of Aβ while up-regulation with behavioral training could induce production of products needed for memory formation. Consistent with that possibility, alongside the APP increase, increased expression of the α-secretase Adam10 was detected in the activated AU profile, potentially biasing the cleavage pattern more towards production sAppα rather than Aβ [23]. This shift could play a dual role of enhancing molecules contributing to memory as well as reducing neurotoxic molecules to serve a neuroprotective function. 

The finding that AU rats appear to more tightly regulate gene expression in basal conditions, relative to both Y and AI, together with a pattern in which considerable numbers of those genes are increased in AU-act rats, is particularly interesting in light of the hyperexcitability of CA3 neurons identified in impaired aged rats [8]. Together, the basal and activated profiles suggest that mechanisms in AU rats are engaged to maintain control over such excitability. Consistent with this interpretation we found increased expression of many genes involved in inhibitory neurotransmission, including GABA and glycine receptor subunits, together with receptor binding partners, and GABA synthetic enzymes in the behaviorally activated AU profile. Increased expression of both GABA synthetic enzymes supports a non-specific increase in GABA production. However, receptor subunit expression increases favor slow inhibitory neurotransmission (Gabbr1 and Gabbr2) and tonic inhibition (Gabra4, Gabra5, Rdx, Nptn) but not synaptic inhibition (Gabra1, Gabra2, Gabrg2, Gphn). Slow, G-protein coupled inhibitory neurotransmission is mediated by GABA-B receptors with each receptor requiring B1 and B2 receptor subunits, both of which are increased in AU activated rats. Activation of GABA-B receptors reduces glutamate release when localized presynaptically and inhibits calcium spikes postsynaptically [31]. GABA-B1 null mice demonstrate a critical role for these receptors in the maintenance of inhibition, where receptor deletion results in hyperexcitability of the CA3 subfield and generalized epilepsy [32]. Both GABA-A α4 and α5 containing receptors mediate tonic inhibition and are localized extrasynaptically where low levels of GABA produce a persistent inhibitory conductance [33]. The anchor protein, radixin, is necessary for the clustering of GABA-A α5 receptors in the membrane, and neuroplastin, a newly identified GABA-A receptor interacting partner, is also associated with extrasynaptic GABA-A α5 subunits [34,35]. The relevance of these activated AU gene increases is supported by a pharmacological targeting study in our aging model, in which administration of a GABA-A α5 positive allosteric modulator improved spatial memory of aged impaired rats [36]. In addition, CA3 specific enhancement of inhibition is sufficient to improve spatial memory performance, supporting the potential cognitive impact of the noted gene changes [12]. Thus, enhanced expression of inhibitory neurotransmission could exert a regulating influence on task induced neuronal activity and reduce the likelihood of the hyperactivity that occurs in AI rats. 

An understanding of the biological basis for preserved cognitive function in the elderly has important implications for appropriate intervention targets in impaired individuals. Much attention to this topic has focused on a disconnection between evidence for brain pathology, in particular signatures of Alzheimer’s disease, and cases where such individuals have been deemed to be clinically normal, with cognitive reserve invoked to account for this discrepancy. Because animals in the outbred rodent model used in the current research are not subject to pathological neurodegenerative disease, the results here are more relevant to the concept of reserve as applied to healthy aging. In that context the construct of reserve is used to account for high performing individuals who do not exhibit the milder deficits that are common in the elderly population. Such reserve might be based on individual differences in brain structure and/or differences in recruitment of neuronal resources within neural networks essential for maintaining cognition. While, studies examining the connectivity and recruitment of neural networks in aged rodents are only just beginning [37] the data presented here support region specific individual differences in recruitment under conditions of behavioral activation as evidenced by differential gene expression patterns, possibly suggesting a reserve-like phenomenon in rodents. 

At the same time, the behavioral capacity, together with studies demonstrating intact encoding properties of neurons in AU rats, have been viewed as providing evidence for maintained brain function on a par with young adults [38]. In the current study, further evidence for maintenance of brain function is provided by the induction of mRNAs with behavioral activation in AU rats that resemble gene expression patterns induced by spatial learning in young adults. Beyond such similarity, however, AU rats differ from young adults, both in basal gene expression profiles as reported previously [14] and, in the current study, for example, in the similarity of AU gene profiles in S and NS task conditions. Indeed, the comparison between S and NS datasets shows a pattern of gene regulation in AU rats that is quite distinct from young rats under the same conditions. It is also notable that the mRNAs contributing to inhibitory function were a particularly distinctive feature of the AU-activated profile, consistent with the interpretation of a coordinated control over neuronal excitability observed in the context of brain aging. Thus, the AU activated profile may reflect selective changes in the context of aging rather than merely maintenance of a young-like brain state. These data reinforce a perspective that preservation of intact memory at older ages may require specific neurobiological adaptations to support the function of critical neuronal circuits and a high level of cognitive performance. Through the use of transcriptome profiling across datasets acquired in a well-characterized model for individual differences in healthy outbred rats here we report evidence for such neuroadaptive aging in rats with preserved cognitive function. These results, based on a gene discovery approach, point to conditions that may better optimize brain function in the context of brain aging. 

## Methods

### Animals

Aged, male Long-Evans rats were obtained at 8-9 mo of age from Charles River Laboratories (Raleigh, NC) and housed in a vivarium at The Johns Hopkins University until 24-26 mo of age for the present study. All rats were individually housed at 25°C and maintained on a 12 hr light/dark cycle. Food and water were provided ad libitum. All rats included in the study were determined to be healthy as confirmed by pathogen-free status throughout the experiments, screening for disability, as well as by necropsies at the time of sacrifice. 

### Ethics statement

All animal procedures were approved by The Johns Hopkins University animal care and use committee (Protocol # RA11A44) in accordance with the National Institutes of Health directive. All efforts were made to minimize animal suffering.

### Behavioral characterization

Behavioral assessment of memory function in a Morris water maze task was conducted as previously described [4]. Briefly, the water maze consisted of a circular pool surrounded by white curtains with black patterns affixed to provide a configuration of spatial cues. The rats were trained for eight days (three trials per day) to locate a camouflaged escape platform that remained at the same location throughout training. Every sixth trial consisted of a probe trial (free swim with no escape platform) that served to assess the development of a spatially localized search for the escape platform. The primary measure used in the spatial learning task was proximity to the escape platform location, a sensitive method for behavioral analysis in this task [39]. A learning index (LI), generated from the proximity measure, was used to define impairment in the rats. Lower scores reflect better performance as they indicate a search closer to the platform location. Aged rats were categorized based on the normative range of young performance established across years of testing with this model. Those aged rats performing within the normative range of young adults were designated aged unimpaired (AU) whereas those performing worse than young were considered aged impaired (AI). Thirty-two animals from 6 separate runs were selected for inclusion in this study to represent a complete range of performance as indicated by learning index and to equate performance across spatial and non-spatial conditions of the induction protocols (Figure 1A). Although young subjects were not used in this study, each run included aged and young subjects to ensure consistency of the background behavioral characterization relative to young adults across runs. 

### Gene expression induction protocol

4-6 weeks after behavioral characterization, all rats were given a single training session (8 trials with 8-min ITI) in a new water maze environment located at a different site as described in detail in [18]. Rats designated for the spatial condition (S protocol) received training in the presence of prominent extramaze spatial cues and in which a visible escape platform remained at the same location for all training trials. The non-spatial group (NS protocol) received training in which the location of the visible platform varied across the trials and the prominent extramaze cues were removed. Intact escape performance during training compared across induction protocols was used to ensure the absence of sensorimotor and motivational deficits that could affect differences in gene expression profiles. One hour after the last training trial, all rats were given 90-sec probe trial without the escape platform. Data were analyzed with a video tracking system (HVS Image Analyzing VP-116) and an IBM PC computer with software developed by HVS Imaging (Hampton, UK).

### Hippocampal dissection

Rats were sacrificed by rapid decapitation immediately after the induction protocol probe trial, and CA3 dissections were performed as previously described [14]. Briefly, the CA3 subfield was microdissected by hand from transverse hippocampal sections under a dissecting microscope. Tissue for each CA3 was pooled across hemispheres of the same animal, frozen on dry ice and then stored at -80°C until all samples were collected. RNA isolated from each animal was hybridized to a separate array. 

### Microarray analysis

RNA samples were sent to the Johns Hopkins Microarray core facility for cRNA labeling and hybridization to Affymetrix rat 230 2.0 microarrays using standard Affymetrix recommended procedures (detailed in [14]). All quality control, normalization, differential expression, and exploratory analysis of microarray data were performed using the open-source R statistical language (http://www.r-project.org/). Raw CEL files from individual hybridizations were imported using the "affy" package in the Bioconductor collection of R packages [40] (http://www.bioconductor.org/). Quality of microarray data was assessed on many levels including RNA degradation plots, boxplots of raw and transformed data and examination of chip pseudoimages. Several dimension reducing algorithms, including principal component analysis (PCA), multidimensional scaling (MDS), and clustering were used to assess variance globally across all expression measures and to identify outlier microarrays. Based on these data, 1 array was excluded from further analysis (1 AI non-spatial subject) resulting in 16 AU arrays (8 spatial and 8 non-spatial) and 15 AI arrays (8 spatial and 7 non-spatial). The "gcrma" package in Bioconductor was used to normalize microarray data following quality control procedures. This involves background correction, quantile normalization, and probeset summarization. All raw data is publicly available via the GEO database http://www.ncbi.nlm.nih.gov/geo/query/acc.cgi?acc=GSE47867.

Significance Analysis in Microarrays d-statistics [41] were used to assess differential expression across groups of animals. This entails a moderated T-statistic, which borrows variance information across genes in order to increase statistical power and to avoid the tendency to find low variance expression changes significant. In addition, an empirically determined low intensity limit was used. This limit was determined by inspecting differential expression statistics across the range of intensity measures. As is common in Affymetrix data normalized with GCRMA, there was a clear level below which differential expression statistics showed markedly different behavior with regards to variance and bias yielding a bimodal distribution of intensities in the log_2_ scale. In this case it was at a value of 4 in the log_2_ scale for all datasets. Expression values below this limit were omitted from the analysis of differential expression of individual genes. Initially spatial and non-spatial arrays were assessed independently, but given the similarity in expression profiles between spatial and non-spatial subjects, these conditions were combined within cognitive groups as described in the main text. To control for false positives introduced by large numbers of comparisons, false discovery rate (FDR) analysis was used to create gene lists containing a precisely estimated proportion of false hits. This is achieved by comparing the observed differential expression statistics to those expected by chance. Those expected by chance are estimated by permuting the group labels of the data many times and recalculating differential expression statistics. For comparisons that included all subjects, i.e. spatial and non-spatial conditions, a 5% FDR was imposed, but this was increased to 10% for comparison between conditions within a cognitive subgroup due to the reduced number of subjects. 

### Multidimensional scaling analysis (MDS)

Using all gene expression measures generated from each microarray exceeding the low expression cut-off, the pair-wise correlation (r) between all possible sample pairs was calculated. An MDS algorithm was used to represent all pair-wise distances (defined to be 1-r) such that each sample was visualized as a single point in 2D space. 

### Functional groups analysis

Functional groups analysis was performed on the list of probesets significantly differentially expressed as determined by SAM between activated AU and activated AI rats. All included probesets were significant at an FDR <0.05. Probesets increased in AU were examined separately from those decreased in AU. Affymetrix identifiers for each probeset in the list were inputted into the free online program DAVID (http://david.abcc.ncifcrf.gov/home.jsp [21,22]) to assess overrepresentation of genes in functional groups. Only functional groups meeting a Bonferroni corrected p<0.05 are included in Table S1. 

### Comparison across gene arrays

The three gene array datasets compared in this study were generated using the same Affymetrix Rat 230 platform. The behaviorally activated and basal RNA were run on the Rat 230-2.0 arrays while the young learning dataset utilized the Rat 230A array, which contained a subset (approximately half) of the probesets employed on the 230-2.0 arrays. In all cases comparisons were made via probeset identifiers, not gene annotations. Because each of these datasets were run independently with different scaling parameters, direct comparison of probeset intensities across datasets was not appropriate. Therefore comparisons were made based on significant gene lists, SAM d-statistics, or fold change values. Although the three microarray experiments were performed independently and should be interpreted accordingly, there was consistency across all experiments regarding animal source and housing conditions, behavioral training procedures, data analysis and apparatus, dissection and RNA extraction protocols, microarray hybridization and microarray analysis. Additionally key personnel were identical across all three studies (RPH, CC and MG). 

### Statistical analysis

Differences in behavioral performance were assessed with analysis of variance (ANOVA) followed by Fisher’s post hoc where appropriate. All statistics are given in the text and/or figure legends. 

## Supporting Information

Figure S1 **Correspondence between spatial and non-spatial CA3 gene expression patterns.** **A**. PCA plot of CA3 gene expression profiles of subjects in the spatial condition. **B**. SAM d-statistic density plot comparing AI-S to AI-NS expression profiles shows no genes differentially expressed between groups at an FDR=0.1. **C**. MDS analysis of AI-S and AI-NS subjects exhibits near complete overlap of subject groups. **D**. SAM d-statistic density plot comparing AU-S to AU-NS expression profiles shows no genes differentially expressed between groups at an FDR=0.1. **E**. MDS analysis of AU-S and AU-NS subjects also exhibits substantial overlap of subject groups.
(PDF)

Figure S2 **Behaviorally activated profiles predominantly differentiate AU from AI rats.** MDS plot of AU-act and AI-act CA3 gene expression profiles.
(PDF)

Figure S3 **Spatial and Non-spatial protocols induce expression profiles distinct from basal expression.** **A**. SAM d-statistics plot comparing the basal aged dataset AU v AI analysis against the corresponding data derived from AU-S v AI-S comparison. **B**. A similar plot of basal AU v AI comparison against the corresponding data derived from the NS data (AU-NS v AI-NS).
(PDF)

Figure S4 **Genes uniquely decreased in AU rats under basal conditions are subsequently increased with behavioral activation in AU-act rats and with spatial learning in young.** **A**. SAM d-statistic comparison between the basal dataset (AU v AI) and behaviorally activated dataset (AU-act v AI-act) is color coded to show AU specific increased (red) and decreased (blue) genes. **B**. SAM d-statistic comparison between the basal dataset (AU v AI) and young spatial learning dataset (Y-LA v Y-CTL) is color coded to show AU specific increased (red) and decreased (blue) genes.
(PDF)

Figure S5 **Relative microarray intensities of GO: Synaptic Transmission functional group genes demonstrate dynamic regulation with behavioral activation.** **A**. Relative gene expression levels from Basal aging dataset. **B**. Relative gene expression levels from behaviorally activated dataset.
(PDF)

Table S1 **DAVID functional groups for AU-activated increased genes.**
(XLSX)

Table S2 **CA3 expressed genes with increased expression in Young learning and AU activated rats.**
(XLSX)

## References

[1] den Dunnen WF, Brouwer WH, Bijlard E, Kamphuis J, van Linschoten K et al. (2008) No disease in the brain of a 115-year-old woman. Neurobiol Aging 29: 1127-1132. doi:10.1016/j.neurobiolaging.2008.04.010. PubMed: 18534718. 18534718

[2] Nyberg L, Lövdén M, Riklund K, Lindenberger U, Bäckman L (2012) Memory aging and brain maintenance. Trends Cogn Sci 16: 292-305. doi:10.1016/j.tics.2012.04.005. PubMed: 22542563. 22542563

[3] Tucker AM, Stern Y (2011) Cognitive reserve in aging. Curr Alzheimer Res 8: 354-360. doi:10.2174/156720511795745320. PubMed: 21222591. 21222591 PMC3135666

[4] Gallagher M, Burwell R, Burchinal M (1993) Severity of spatial learning impairment in aging: development of a learning index for performance in the Morris water maze. Behav Neurosci 107: 618-626. doi:10.1037/0735-7044.107.4.618. PubMed: 8397866. 8397866

[5] Albert MS (2011) Changes in cognition. Neurobiol Aging 32 (Suppl 1): S58-S63. doi:10.1016/j.neurobiolaging.2011.09.010. PubMed: 22078174. 22078174 PMC3929949

[6] Wilson IA, Gallagher M, Eichenbaum H, Tanila H (2006) Neurocognitive aging: prior memories hinder new hippocampal encoding. Trends Neurosci 29: 662-670. doi:10.1016/j.tins.2006.10.002. PubMed: 17046075. 17046075 PMC2614702

[7] Smith TD, Adams MM, Gallagher M, Morrison JH, Rapp PR (2000) Circuit-specific alterations in hippocampal synaptophysin immunoreactivity predict spatial learning impairment in aged rats. J Neurosci 20: 6587-6593. PubMed: 10964964. 10964964 10.1523/JNEUROSCI.20-17-06587.2000 PMC6772954

[8] Wilson IA, Ikonen S, Gallagher M, Eichenbaum H, Tanila H (2005) Age-associated alterations of hippocampal place cells are subregion specific. J Neurosci 25: 6877-6886. doi:10.1523/JNEUROSCI.1744-05.2005. PubMed: 16033897. 16033897 PMC6725350

[9] Yassa MA, Stark SM, Bakker A, Albert MS, Gallagher M et al. (2010) High-resolution structural and functional MRI of hippocampal CA3 and dentate gyrus in patients with amnestic Mild Cognitive Impairment. Neuroimage 51: 1242-1252. doi:10.1016/j.neuroimage.2010.03.040. PubMed: 20338246. 20338246 PMC2909476

[10] Yassa MA, Lacy JW, Stark SM, Albert MS, Gallagher M et al. (2011) Pattern separation deficits associated with increased hippocampal CA3 and dentate gyrus activity in nondemented older adults. Hippocampus 21: 968-979. PubMed: 20865732. 20865732 10.1002/hipo.20808 PMC3010452

[11] Bakker A, Krauss GL, Albert MS, Speck CL, Jones LR et al. (2012) Reduction of hippocampal hyperactivity improves cognition in amnestic mild cognitive impairment. Neuron 74: 467-474. doi:10.1016/j.neuron.2012.03.023. PubMed: 22578498. 22578498 PMC3351697

[12] Koh MT, Haberman RP, Foti S, McCown TJ, Gallagher M (2010) Treatment strategies targeting excess hippocampal activity benefit aged rats with cognitive impairment. Neuropsychopharmacology 35: 1016-1025. doi:10.1038/npp.2009.207. PubMed: 20032967. 20032967 PMC2820138

[13] Sanchez PE, Zhu L, Verret L, Vossel KA, Orr AG et al. (2012) Levetiracetam suppresses neuronal network dysfunction and reverses synaptic and cognitive deficits in an Alzheimer's disease model. Proc Natl Acad Sci U S A. 10.1073/pnas.1121081109 PMC3479491 22869752

[14] Haberman RP, Colantuoni C, Stocker AM, Schmidt AC, Pedersen JT et al. (2011) Prominent hippocampal CA3 gene expression profile in neurocognitive aging. Neurobiol Aging 32: 1678-1692. doi:10.1016/j.neurobiolaging.2009.10.005. PubMed: 19913943. 19913943 PMC2891610

[15] Guzowski JF, Lyford GL, Stevenson GD, Houston FP, McGaugh JL et al. (2000) Inhibition of activity-dependent arc protein expression in the rat hippocampus impairs the maintenance of long-term potentiation and the consolidation of long-term memory. J Neurosci 20: 3993-4001. PubMed: 10818134. 10818134 10.1523/JNEUROSCI.20-11-03993.2000 PMC6772617

[16] Guzowski JF, Setlow B, Wagner EK, McGaugh JL (2001) Experience-dependent gene expression in the rat hippocampus after spatial learning: a comparison of the immediate-early genes Arc, c-fos, and zif268. J Neurosci 21: 5089-5098. PubMed: 11438584. 11438584 10.1523/JNEUROSCI.21-14-05089.2001 PMC6762831

[17] Igaz LM, Vianna MR, Medina JH, Izquierdo I (2002) Two time periods of hippocampal mRNA synthesis are required for memory consolidation of fear-motivated learning. J Neurosci 22: 6781-6789. PubMed: 12151558. 12151558 10.1523/JNEUROSCI.22-15-06781.2002 PMC6758123

[18] Haberman RP, Lee HJ, Colantuoni C, Koh MT, Gallagher M (2008) Rapid encoding of new information alters the profile of plasticity-related mRNA transcripts in the hippocampal CA3 region. Proc Natl Acad Sci U S A 105: 10601-10606. doi:10.1073/pnas.0804292105. PubMed: 18650386. 18650386 PMC2492487

[19] Gallagher M, Burwell RD (1989) Relationship of age-related decline across several behavioral domains. Neurobiol Aging 10: 691-708. doi:10.1016/0197-4580(89)90006-7. PubMed: 2628781. 2628781

[20] Robitsek RJ, Fortin NJ, Koh MT, Gallagher M, Eichenbaum H (2008) Cognitive aging: a common decline of episodic recollection and spatial memory in rats. J Neurosci 28: 8945-8954. doi:10.1523/JNEUROSCI.1893-08.2008. PubMed: 18768688. 18768688 PMC2585597

[21] Huang da W, Sherman BT, Lempicki RA (2009) Systematic and integrative analysis of large gene lists using DAVID bioinformatics resources. Nat Protoc 4: 44-57. PubMed: 19131956. 19131956 10.1038/nprot.2008.211

[22] Huang da W, Sherman BT, Lempicki RA (2009) Bioinformatics enrichment tools: paths toward the comprehensive functional analysis of large gene lists. Nucleic Acids Res 37: 1-13. doi:10.1093/nar/gkp505. PubMed: 19033363. 19033363 PMC2615629

[23] Kim D, Tsai LH (2009) Bridging physiology and pathology in AD. Cell 137: 997-1000. doi:10.1016/j.cell.2009.05.042. PubMed: 19524503. 19524503

[24] Gallagher M, Colantuoni C, Eichenbaum H, Haberman RP, Rapp PR et al. (2006) Individual differences in neurocognitive aging of the medial temporal lobe. AGE: Journal of the American Aging Association 28: 221-223. doi:10.1007/s11357-006-9017-5. PMC3259151 22253491

[25] Yassa MA, Mattfeld AT, Stark SM, Stark CE (2011) Age-related memory deficits linked to circuit-specific disruptions in the hippocampus. Proc Natl Acad Sci U S A 108: 8873-8878. doi:10.1073/pnas.1101567108. PubMed: 21555581. 21555581 PMC3102362

[26] Cavallaro S, D'Agata V, Manickam P, Dufour F, Alkon DL (2002) Memory-specific temporal profiles of gene expression in the hippocampus. Proc Natl Acad Sci U S A 99: 16279-16284. doi:10.1073/pnas.242597199. PubMed: 12461180. 12461180 PMC138602

[27] Boric K, Munoz P, Gallagher M, Kirkwood A (2008) Potential adaptive function for altered long-term potentiation mechanisms in aging hippocampus. J Neurosci 28: 8034-8039. doi:10.1523/JNEUROSCI.2036-08.2008. PubMed: 18685028. Available online at: 10.1523/JNEUROSCI.2036-08.2008 Available online at: PubMed: 18685028 18685028 PMC2615232

[28] Lee HK, Min SS, Gallagher M, Kirkwood A (2005) NMDA receptor-independent long-term depression correlates with successful aging in rats. Nat Neurosci 8: 1657-1659. doi:10.1038/nn1586. PubMed: 16286930. 16286930

[29] Yang S, Megill A, Ardiles AO, Ransom S, Tran T et al. (2013) Integrity of mGluR-LTD in the Associative/Commissural Inputs to CA3 Correlates with Successful Aging in Rats. J Neurosci 33: 12670-12678. doi:10.1523/JNEUROSCI.1086-13.2013. PubMed: 23904603. 23904603 PMC3728684

[30] Parihar MS, Brewer GJ (2010) Amyloid-beta as a modulator of synaptic plasticity. J Alzheimers Dis 22: 741-763. PubMed: 20847424. 20847424 10.3233/JAD-2010-101020 PMC3079354

[31] Chalifoux JR, Carter AG (2011) GABAB receptor modulation of synaptic function. Curr Opin Neurobiol 21: 339-344. doi:10.1016/j.conb.2011.02.004. PubMed: 21376567. 21376567 PMC3092847

[32] Brown JT, Gill CH, Farmer CE, Lanneau C, Randall AD et al. (2003) Mechanisms contributing to the exacerbated epileptiform activity in hippocampal slices of GABAB1 receptor subunit knockout mice. Epilepsy Res 57: 121-136. doi:10.1016/j.eplepsyres.2003.10.013. PubMed: 15013053. 15013053

[33] Hines RM, Davies PA, Moss SJ, Maguire J (2012) Functional regulation of GABAA receptors in nervous system pathologies. Curr Opin Neurobiol 22: 552-558. doi:10.1016/j.conb.2011.10.007. PubMed: 22036769. 22036769 PMC3846183

[34] Loebrich S, Bähring R, Katsuno T, Tsukita S, Kneussel M (2006) Activated radixin is essential for GABAA receptor alpha5 subunit anchoring at the actin cytoskeleton. EMBO J 25: 987-999. doi:10.1038/sj.emboj.7600995. PubMed: 16467845. 16467845 PMC1409722

[35] Sarto-Jackson I, Milenkovic I, Smalla KH, Gundelfinger ED, Kaehne T et al. (2012) The cell adhesion molecule neuroplastin-65 is a novel interaction partner of gamma-aminobutyric acid type A receptors. J Biol Chem 287: 14201-14214. doi:10.1074/jbc.M111.293175. PubMed: 22389504. 22389504 PMC3340160

[36] Koh MT, Rosenzweig-Lipson S, Gallagher M (2013) Selective GABA(A) alpha5 positive allosteric modulators improve cognitive function in aged rats with memory impairment. Neuropharmacology 64: 145-152. doi:10.1016/j.neuropharm.2012.06.023. PubMed: 22732440. 22732440 PMC3445657

[37] Lu H, Zou Q, Gu H, Raichle ME, Stein EA et al. (2012) Rat brains also have a default mode network. Proc Natl Acad Sci U S A 109: 3979-3984. doi:10.1073/pnas.1200506109. PubMed: 22355129. 22355129 PMC3309754

[38] Wilson IA, Ikonen S, McMahan RW, Gallagher M, Eichenbaum H et al. (2003) Place cell rigidity correlates with impaired spatial learning in aged rats. Neurobiol Aging 24: 297-305. doi:10.1016/S0197-4580(02)00080-5. PubMed: 12498963. 12498963

[39] Maei HR, Zaslavsky K, Teixeira CM, Frankland PW (2009) What is the Most Sensitive Measure of Water Maze Probe Test Performance? Front Integr Neurosci 3: 4 PubMed: 19404412. 19404412 10.3389/neuro.07.004.2009 PMC2659169

[40] Irizarry RA, Hobbs B, Collin F, Beazer-Barclay YD, Antonellis KJ et al. (2003) Exploration, normalization, and summaries of high density oligonucleotide array probe level data. Biostatistics 4: 249-264. doi:10.1093/biostatistics/4.2.249. PubMed: 12925520. 12925520

[41] Tusher VG, Tibshirani R, Chu G (2001) Significance analysis of microarrays applied to the ionizing radiation response. Proc Natl Acad Sci U S A 98: 5116-5121. doi:10.1073/pnas.091062498. PubMed: 11309499. 11309499 PMC33173

