# Supplementary material for: Behaviorally Activated mRNA Expression Profiles Produce Signatures of Learning and Enhanced Inhibition in Aged Rats with Preserved Memory
Source: PLoS One. 2013 Dec 13;8(12):e83674. doi: 10.1371/journal.pone.0083674 (PMC3862806; doi:10.1371/journal.pone.0083674)
Supplement: Figure S1 — Correspondence between spatial and non-spatial CA3 gene expression patterns. A. PCA plot of CA3 gene expression profiles of subjects in the spatial condition. B. SAM d-statistic density plot comparing AI-S to AI-NS expression profiles shows no genes differentially expressed between groups at an FDR=0.1. C. MDS analysis of AI-S and AI-NS subjects exhibits near complete overlap of subject groups. D. SAM d-statistic density plot comparing AU-S to AU-NS expression profiles shows no genes differentially expressed between groups at an FDR=0.1. E. MDS analysis of AU-S and AU-NS subjects also exhibits substantial overlap of subject groups. (PDF) [file pone.0083674.s001.pdf]

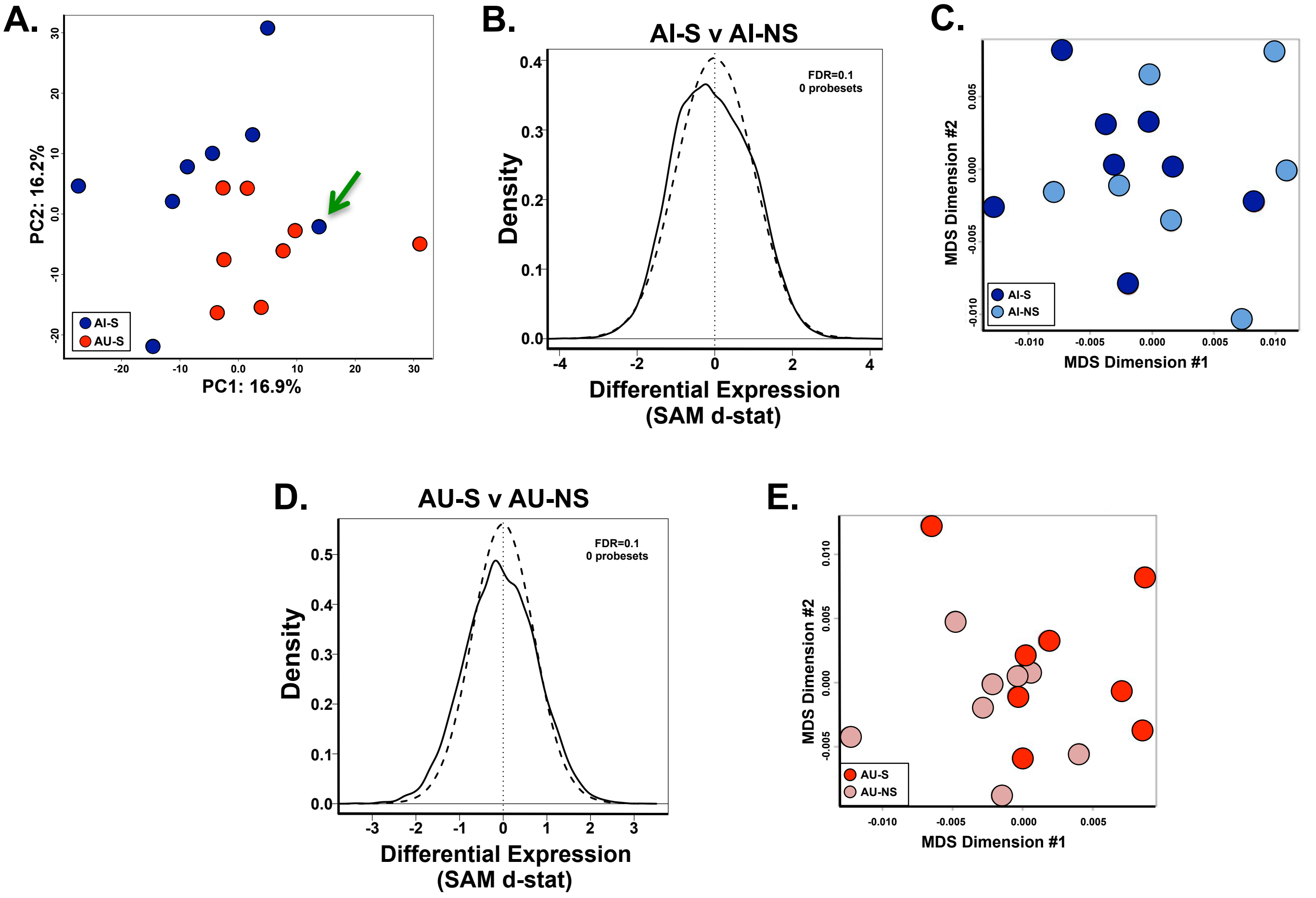

Figure S1: Correspondence between spatial and non-spatial CA3 gene expression patterns. A. PCA plot of CA3 gene expression profiles of rats in the spatial condition. Each point within the graph represents a single array colored by cognitive phenotype as indicated. The distance between points is an indication of relative similarity of mRNA profiles. The amount of variance accounted for in each component is noted on the axes. Similar to MDS, AU-S arrays (red) cluster together partially segregated from AI-S arrays (blue).The green arrow points to an AI rat with a borderline LI (LI=254). **B.** SAM d-statistic density plots comparing AI-S to AI-NS expression profiles show no genes differentially expressed between groups at an FDR=0.1. The black dashed line represents the expected, random d-statistic density distribution while the back curve represents the distribution observed in the comparison between AI-S and AI-NS. Positive d-statistic values represent increased expression in AI-S. **C.** MDS analysis of AI-S and AI-NS subjects show near complete overlap of subject groups. **D.** SAM d-statistic density plots comparing AU-S to AU-NS expression profiles show no genes differentially expressed between groups at an FDR=0.1. The black dashed line represents the expected, random d-statistic density distribution while the back curve represents the distribution observed in the comparison between AU-S and AU-NS. Positive d-statistic values represent increased expression in AU-S. **E.** MDS analysis of AU-S and AU-NS subjects also shows substantial overlap of subject groups.
