# Supplementary material for: Behaviorally Activated mRNA Expression Profiles Produce Signatures of Learning and Enhanced Inhibition in Aged Rats with Preserved Memory
Source: PLoS One. 2013 Dec 13;8(12):e83674. doi: 10.1371/journal.pone.0083674 (PMC3862806; doi:10.1371/journal.pone.0083674)
Supplement: Figure S2 — Behaviorally activated profiles predominantly differentiate AU from AI rats. MDS plot of AU-act and AI-act CA3 gene expression profiles. (PDF) [file pone.0083674.s002.pdf]

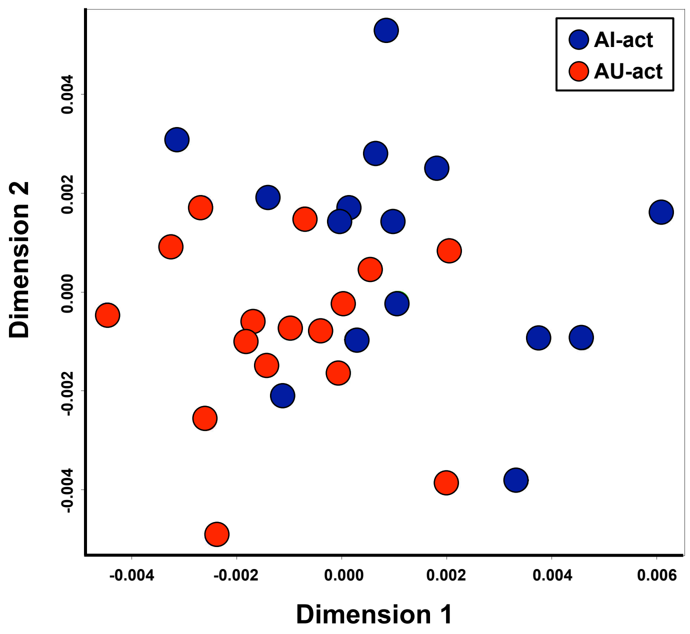

Figure S2: Behaviorally activated profiles predominantly differentiate AU from AI rats. MDS plot of AU-act and AI-act CA3 gene expression profiles. Each point within the graph represents a single array colored by cognitive phenotype as indicated. The distance between points is an indication of relative similarity of mRNA profiles. Similar to MDS with the spatial condition alone, AU arrays (red) cluster together partially segregated from AI arrays (blue).
