# Supplementary material for: Behaviorally Activated mRNA Expression Profiles Produce Signatures of Learning and Enhanced Inhibition in Aged Rats with Preserved Memory
Source: PLoS One. 2013 Dec 13;8(12):e83674. doi: 10.1371/journal.pone.0083674 (PMC3862806; doi:10.1371/journal.pone.0083674)
Supplement: Figure S3 — Spatial and Non-spatial protocols induce expression profiles distinct from basal expression. A. SAM d-statistics plot comparing the basal aged dataset AU v AI analysis against the corresponding data derived from AU-S v AI-S comparison. B. A similar plot of basal AU v AI comparison against the corresponding data derived from the NS data (AU-NS v AI-NS). (PDF) [file pone.0083674.s003.pdf]

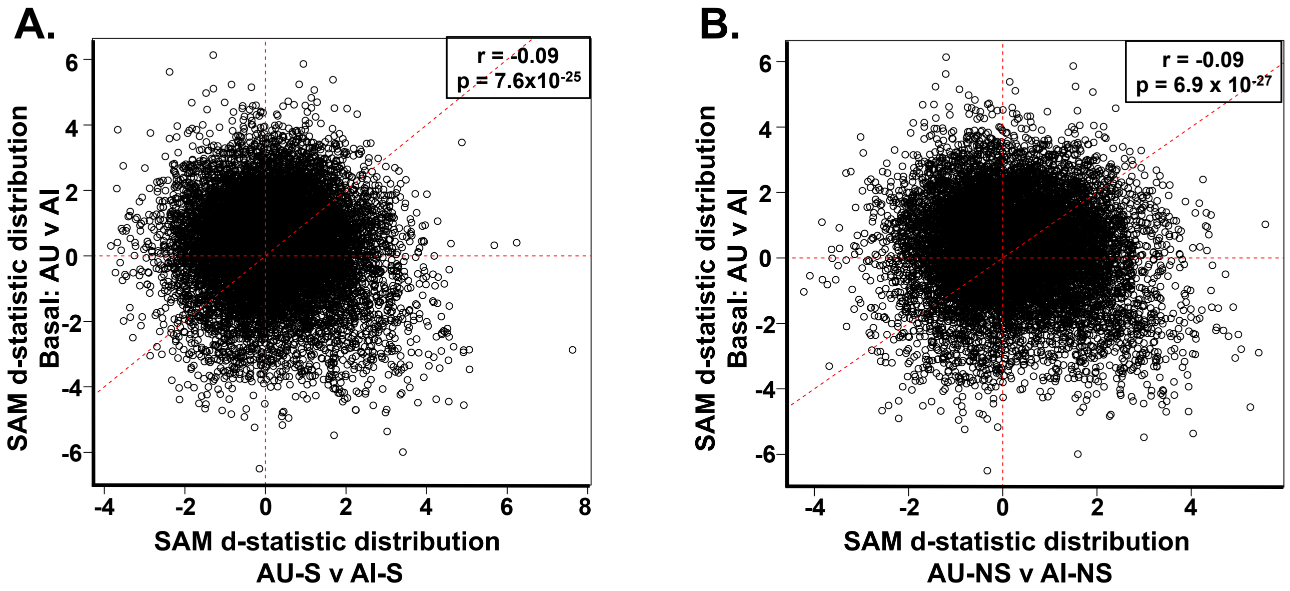

Figure S3. **Spatial and Non-spatial protocols induce expression profiles distinct from basal expression**. **A.** SAM d-statistics for each probeset derived from basal aged dataset AU v AI comparison [14] are plotted on the Y-axis against the corresponding data derived from AU-S v AI-S comparison on the X-axis. A slight negative correlation was found ($r=-0.09$; $p=7.6 \times 10^{-25}$) that was significant only because of the large number of data points(N>15,000 probesets). The lack of positive correlation indicated little similarity between the spatial and basal profiles. **B.** SAM d-statistics for each probeset derived from basal aged dataset AU v AI comparison are plotted on the Y-axis against the corresponding data derived from the NS data (AU-NS v AI-NS) on the X-axis. A slight negative correlation was found ($r=-0.09$; $p=6.9 \times 10^{-27}$) that was significant only because of the large number of data points(N>15,000 probesets). The lack of positive correlation indicated little similarity between the NS and basal profiles.
