# Supplementary material for: Behaviorally Activated mRNA Expression Profiles Produce Signatures of Learning and Enhanced Inhibition in Aged Rats with Preserved Memory
Source: PLoS One. 2013 Dec 13;8(12):e83674. doi: 10.1371/journal.pone.0083674 (PMC3862806; doi:10.1371/journal.pone.0083674)
Supplement: Figure S4 — Genes uniquely decreased in AU rats under basal conditions are subsequently increased with behavioral activation in AU-act rats and with spatial learning in young. A. SAM d-statistic comparison between the basal dataset (AU v AI) and behaviorally activated dataset (AU-act v AI-act) is color coded to show AU specific increased (red) and decreased (blue) genes. B. SAM d-statistic comparison between the basal dataset (AU v AI) and young spatial learning dataset (Y-LA v Y-CTL) is color coded to show AU specific increased (red) and decreased (blue) genes. (PDF) [file pone.0083674.s004.pdf]

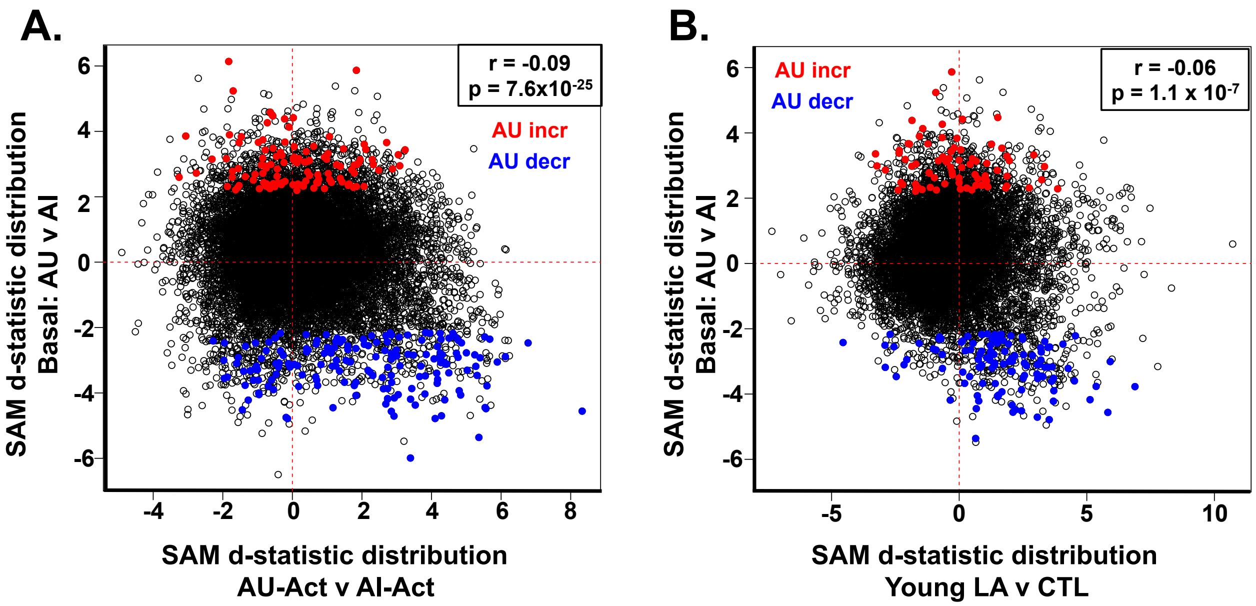

Figure S4. **Genes uniquely decreased in AU rats under basal conditions are subsequently increased with behavioral activation in AU-act rats and with spatial learning in young**. **A.** SAM d-statistic comparison between the basal dataset (AU v AI) and behaviorally activated dataset (AU-act v AI-act) is shown as in Fig 4. Each dot represents a single probeset, which have been color coded to show AU specific increased (red) and decreased (blue) genes. The preponderance of AU decreased genes, defined in the text as being differentially expressed relative to both young and AI under basal conditions are increased in AU-act rats relative to AI-act. **B.** SAM d-statistic comparison between the basal dataset (AU v AI) and young spatial learning dataset (Y-LA v Y-CTL) is shown. Each dot represents a single probeset, which have been color coded to show AU specific increased (red) and decreased (blue) genes. The preponderance of AU decreased genes are increased Y-LA rats relative to control.
