# Supplementary material for: Behaviorally Activated mRNA Expression Profiles Produce Signatures of Learning and Enhanced Inhibition in Aged Rats with Preserved Memory
Source: PLoS One. 2013 Dec 13;8(12):e83674. doi: 10.1371/journal.pone.0083674 (PMC3862806; doi:10.1371/journal.pone.0083674)
Supplement: Figure S5 — Relative microarray intensities of GO: Synaptic Transmission functional group genes demonstrate dynamic regulation with behavioral activation. A. Relative gene expression levels from Basal aging dataset. B. Relative gene expression levels from behaviorally activated dataset. (PDF) [file pone.0083674.s005.pdf]

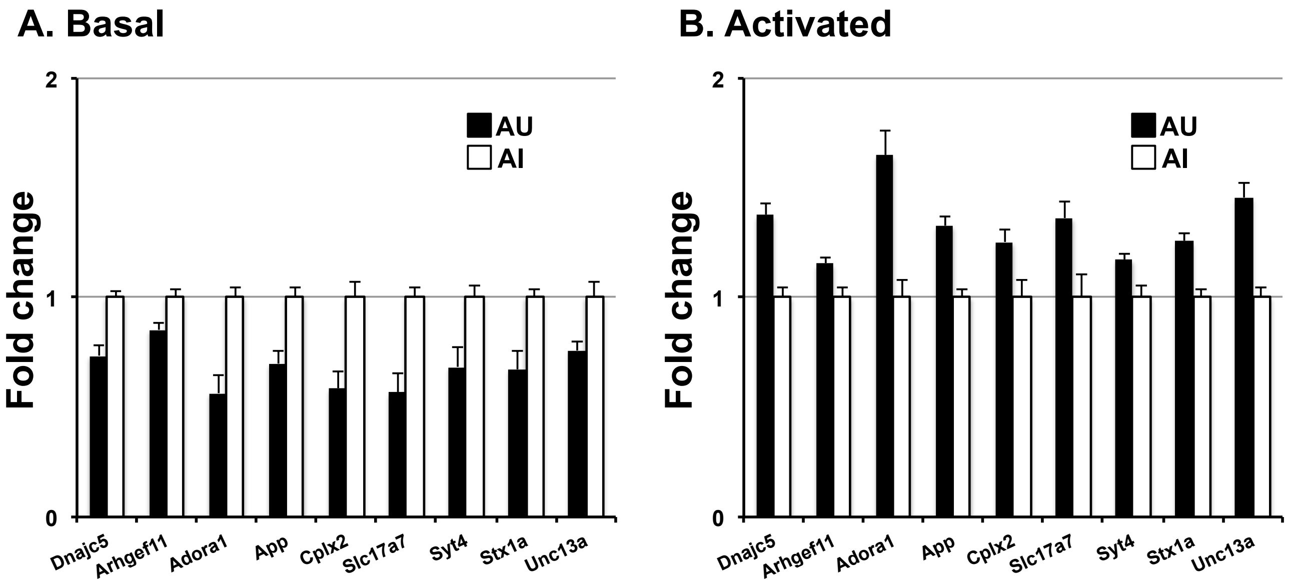

Figure S5. Relative microarray intensities of GO: Synaptic Transmission functional group genes demonstrate dynamic regulation with behavioral activation. A. Relative microarray expression intensities from from Basal aging dataset from the overlap of AU-specific decreased genes and activated AU increased genes as described in the text. **B.** Relative gene expression levels of the same genes in behaviorally activated dataset. All intensities normalized to AI subjects. Error bars indicate SEM. All differences significant at $p<0.05$ for **A.** and FDR<0.05 for **B.** Abbreviations: Dnajc5, DnaJ (Hsp40) homolog, subfamily C, member 5; Arhgef11, Rho guanine nucleotide exchange factor (GEF) 11; Adora1, adenosine A1 receptor; App, amyloid beta (A4) precursor protein; Cplx2, complexin 2, Slc17a7, solute carrier family 17 (sodium-dependent inorganic phosphate cotransporter), member 7; Syt4, synaptotagmin IV; Stx1a, syntaxin 1A (brain), Unc13a, unc-13 homolog A (C. elegans).
